# GLT-1 Knockdown Inhibits Ceftriaxone-Mediated Improvements on Cognitive Deficits, and GLT-1 and xCT Expression and Activity in APP/PS1 AD Mice

**DOI:** 10.3389/fnagi.2020.580772

**Published:** 2020-10-06

**Authors:** JunXia Gao, LiZhe Liu, Chao Liu, ShuJuan Fan, LiRong Liu, ShuFeng Liu, Xiao-Hui Xian, Wen-Bin Li

**Affiliations:** ^1^Department of Pathophysiology, Neuroscience Research Center, Hebei Medical University, Shijiazhuang, China; ^2^Hebei Key Lab of Laboratory Animal Science, Laboratory Animal Center, Hebei Medical University, Shijiazhuang, China

**Keywords:** ceftriaxone, GLT-1, GLT-1 knockdown, glutamate uptake, xCT, glutathione level, APP/PS1 mice

## Abstract

**Objective:**

Glutamate transporter-1 (GLT-1) and system x_*c*_^–^ mediate glutamate uptake and release, respectively. Ceftriaxone has been reported to upregulate GLT-1 expression and improve cognitive decline in APP/PS1 mice. The aim of the present study was to elucidate the role of GLT-1 in ceftriaxone-mediated improvement on cognitive deficits and associated changes in xCT (catalytic subunit of system x_*c*_^–^) expression and activity using GLT-1 knockdown APP/PS1 mice.

**Methods:**

GLT-1 knockdown (GLT-1^±^) mice were generated in C57BL/6J mice using the CRISPR/Cas9 technique and crossed to APP/PS1 mice to generate GLT-1^±^APP/PS1 mice. The cognition was evaluated by novel object recognition and Morris water maze tests. GLT-1 and xCT expression, GLT-1 uptake for glutamate, and glutathione levels of hippocampus were assayed using Western blot and immunohistochemistry, ^3^H-glutamate, and glutathione assay kit, respectively.

**Results:**

In comparison with wild-type mice, APP/PS1 mice exhibited significant cognitive deficits, represented with poor performance in novel object recognition and Morris water maze tests, downregulated GLT-1 expression and glutamate uptake. Ceftriaxone treatment significantly improved the above impairments in APP/PS1 mice, but had negligible impact in GLT-1^±^APP/PS1 mice. The xCT expression increased in APP/PS1 and GLT-1^±^APP/PS1 mice. This upregulation might be a compensatory change against the accumulated glutamate resulting from GLT-1 impairment. Ceftriaxone treatment restored xCT expression in APP/PS1 mice, but not in GLT-1^±^APP/PS1 mice. Glutathione levels decreased in APP/PS1 mice in comparison to the wild-type group. After ceftriaxone administration, the decline in glutathione level was restored in APP/PS1 mice, but not in GLT-1^±^APP/PS1 mice.

**Conclusion:**

Ceftriaxone improves cognitive impairment of APP/PS1 mice by upregulating GLT-1-mediated uptake of glutamate and co-regulation of GLT-1 and xCT in APP/PS1 mice.

## Introduction

Alzheimer’s disease (AD) is a common age-related neurodegenerative disease with learning and memory impairment, and progressive dementia (Selkoe, 2002). Although the exact pathogenesis of AD has not been fully elucidated, numerous studies have shown that dysfunction of the glutamatergic neuronal system plays an important role in the pathogenesis of AD (Hardy et al., 1987; Masliah et al., 2000; Hynd et al., 2004; Scott et al., 2011). Glutamate, the primary neurotransmitter of glutamatergic neuronal system, exerts an essential function in learning and memory (Willard and Koochekpour, 2013). Either dysfunction in glutamate reutilization or overaccumulation of glutamate in extracellular space in the brain, resulting in excitotoxicity and leading to synapse loss, would impair synaptic transmission and connection, and thus induce cognitive decline (Francis, 2003; Hynd et al., 2004; Talantova et al., 2013; Audrain et al., 2016). The concentration of glutamate in the synaptic cleft is principally regulated by glutamate transporters, especially glutamate transporter-1 (GLT-1), which accounts for a major role in glutamate uptake and exerts an important role in reutilization of glutamate as a neurotransmitter and prevention of glutamate excitotoxicity (Haugeto et al., 1996; Rothstein et al., 1996). Impairment of GLT-1 expression and/or uptake and the subsequent dysregulation in glutamate level exerts a crucial role in the pathogenesis of AD. For example, amyloid-peptide (Aβ) reduces glutamate uptake by decreasing GLT-1 expression or inducing mislocalization and endocytosis of GLT-1 in astrocytes of AD model animals (Scimemi et al., 2013; Tong et al., 2017). Moreover, GLT-1 expression in the hippocampus of 3xTg-AD model mouse was found to be significantly downregulated (Zumkehr et al., 2015). Glutamate uptake activity in astrocytes derived from the cortex of patients with AD is also significantly reduced (Liang et al., 2002; Scott et al., 2011). Transgenic AD mice with GLT-1 knockdown show aggravated cognitive impairment (Mookherjee et al., 2011). Moreover, dihydrokainic acid, a GLT-1 inhibitor, impairs memory performance in rat and mouse (Bechtholt-Gompf et al., 2010; Tian et al., 2019). The reports suggest that regulation of GLT-1 expression and uptake activity may be protective against cognitive deficits in AD.

Ceftriaxone (Cef), a β-lactam antibiotic, has been reported to increase GLT-1 expression significantly, and this increase can provide neuroprotection in animal models of amyotrophic lateral sclerosis (Rothstein et al., 2005), Parkinson’s disease (Hsu et al., 2015; Hsieh et al., 2017), brain ischemic preconditioning (Chu et al., 2007; Harvey et al., 2011; Hu et al., 2015; Krzyzanowska et al., 2017), pain (Hu et al., 2010), and seizure (Soni et al., 2015; Hussein et al., 2016). Recently, we have reported that Cef can improve learning and memory impairment in early stage APP/PS1 AD mouse, accompanied by an upregulation of GLT-1 expression (Fan et al., 2018). The finding suggests a possibility that GLT-1 upregulation mediated the above effect after Cef treatment. Here, to prove the possibility, we used partial GLT-1 knockout APP/PS1 (GLT-1^±^APP/PS1) AD mouse to investigate the influence of Cef on learning and memory deficits by novel object recognition and Morris water maze tests, GLT-1 expression, and glutamate uptake capacity of the AD mice.

System x_*c*_^–^ is a cystine/glutamate antiporter, which imports cystine in exchange for intracellular glutamate (Bannai and Kitamura, 1980; Lo et al., 2008; Lewerenz et al., 2013). The exchange promotes the synthesis of glutathione and increases antioxidant activity in astrocytes while simultaneously participating in the maintenance of glutamate homeostasis. The activity of the cystine/glutamate antiporter is stimulated by an increased activity of glutamate transporter (Rimaniol et al., 2001; Lewerenz et al., 2006). So, the present study also aimed to observe changes in xCT expression (the catalytic subunit of system x_*c*_^–^) and levels of glutathione, to reflect the activity of System x_*c*_^–^ in GLT-1^±^APP/PS1 AD mouse, and the influence of Cef on its expression and activity.

## Materials and Methods

### Experimental Animals, Grouping, and Protocols

Ninety-seven male mice aged 6 months old (25–30 g in weight) were used. The mice were bred in the animal facilities of the Laboratory Animal Center of Hebei Medical University, China. The animals were housed in a temperature-controlled facility, at 23°C, with a 12-h light/dark cycle and free access to food and water. All water, food, and padding used in the cages were sterilized by autoclaving. All experimental procedures were performed according to the “ARRIVE” guidelines (McGrath and Lilley, 2015) and approved by the Animal Care and Use Committee of the Hebei Medical University, China. All efforts were done to minimize suffering and numbers of mice.

**FIGURE 1:**
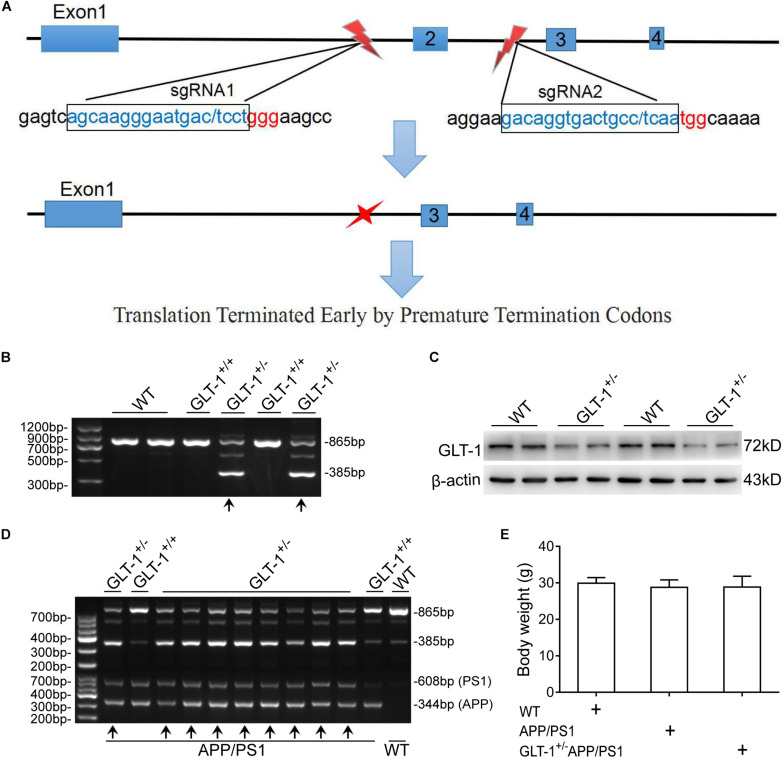
The generation and identification of glutamate transporter-1 (GLT-1)^±^APP/PS1 mice. **(A)** Schematic of the genetic modification technique used. SgRNA1 and sgRNA2 were designed as shown. One incision was made on each side of the second exon to knock out a 480 -bp sequence, which includes the second exon. This modification induces transcoding mutations of GLT-1 that terminate prematurely. **(B)** PCR-based genotyping of mice offspring using tail tissue showing genotype of GLT-1 knockdown (the bands have been indicated by upward arrows). **(C)** Western blot analysis showing downregulated GLT-1 expression in the hippocampus of GLT-1^±^ mice compared with wild-type mice. **(D)** PCR-based genotype identification of the GLT-1^±^APP/PS1 mice offspring, clearly showing GLT-1 knockdown and APP/PS1 transgenic features (bands have been indicated by upward arrows). **(E)** There is no difference in the body weight of wild-type, APP/PS1, and GLT-1^±^APP/PS1 mice at 6 months old.

The animals used in the present work include wild-type (C57BL/6J), APP/PS1, and GLT-1^±^APP/PS1 mice. The APP/PS1 mice were purchased from the Chinese Academy of Medical Sciences. This mouse overexpresses human amyloid precursor protein harboring the Swedish (K594M/N595L) mutation and presenilin 1 deleted in exon 9 in a C57BL/6J genetic background, and shows pathological phenotypes of AD, including learning and memory impairment at 3–5 months of age and several senile plaques at 12 months of age (Webster et al., 2014). The GLT-1^±^APP/PS1 mouse was obtained by crossing the APP/PS1 and GLT-1^±^ mice, as described previously (Mookherjee et al., 2011). Briefly, the GLT-1^±^ mouse was first generated in a C57BL/6J mouse using the CRISPR/Cas9 technique. The NGG in the PAM locus of *slcla2* (reference sequence: NC_000068.7) was selected for designing sgRNA. sgRNA1 (GCAAGGGAATGACTCCTGGGAA) and sgRNA2 (ACAGGTGCCTCAATGGCA) were selected from the upstream and downstream regions of the second exon, respectively. A 480-bp sequence, containing the shared second exon, was knocked-out, which resulted in transcoding mutations and premature termination (Figure 1). After screening and sequencing, the GLT-1^±^ mouse, with reduced GLT-1 expression levels, was obtained. The GLT-1^±^ mouse has a normal life span compared to the wild-type mouse (Tanaka, 1997). Thereafter, the GLT-1^±^ mouse was crossed with the transgenic APP/PS1 mouse to generate the GLT-1^±^APP/PS1 mouse. The gene-modified pups were identified by PCR using DNA extracted from the tail tissue of the mouse at 3 weeks of age. Only mice that showed clear APPswe/PS1dE9 transgenic features and GLT-1^±^ knockdown features (Figure 1) were used in the study.

The study designed included wild-type (C57BL/6J), APP/PS1, GLT-1^±^APP/PS1, Cef + APP/PS1, and Cef + GLT-1^±^APP/PS1 groups. The mice in Cef + APP/PS1 and Cef + GLT-1^±^APP/PS1 groups were administrated Cef by peritoneal injection for 14 days, once a day at a dose of 200 mg/kg, which was determined according to previous reports (Rothstein et al., 2005; Hu et al., 2015; Fan et al., 2018). The mice in other groups were administrated normal saline as solvent control, using the same protocols. After completion of the administration, all mice went through behavioral tests. Novel object recognition and Morris water maze tests are common methods in evaluating cognitive deficits of AD animals in non-spatial working memory ability and spatial working memory task (Morris et al., 1982; Vorhees and Williams, 2014; Balderas et al., 2015). Thus, the mice in the present study received novel object recognition and Morris water maze tests separately. After completing the behavioral test, the mice were euthanized, and the hippocampi were collected under anesthesia for the assays of GLT-1 and xCT expression, GLT-1 uptake, and GSH level. Details of the animal number for each test are shown in Table 1.

**TABLE 1:** The details of the animal number in each test.

Group	Number of animals in each test						
	NOR	MWM	GLT-1 exp		GLT-1 uptake	xCT (WB)	GSH level
			WB	IHC			
Wild type	8	10	6	6	6	6	6
APP/PS1	9	13	6	6	6	6	6
GLT-1^±^APP/PS1	8	10	6	6	6	6	6
Cef + APP/PS1	8	12	6	6	6	6	6
Cef + GLT-1^±^APP/PS1	6	10	6	6	6	6	6

*NOR, novel objective recognition test; MWM, Morris water maze test; exp, expression; WB, western blot; IHC, immunohistochemistry; GSH, glutathione; GLT-1, glial glutamate transporter-1; xCT, catalytic subunit of system x_*c*_^–^; Cef, Ceftriaxone. WB for GLT-1 and xCT expression and the assay of GSH level were performed using the same six animals, from which, the right hippocampus was used for WB of GLT-1 and xCT expression, and the left hippocampus was used for the assay GSH level. The animals subjected to the assays of GLT-1 expression and uptake, xCT expression and GSH level received behavioral tests of which three animals received the NOR test, and three received the MWM test, except three animals in the assay of GLT-1 uptake in Cef + GLT-1^±^APP/PS1 group did not receive the NOR test, because of trouble of the equipment.*

### PCR for Identification of Gene Modification

The specific primers used were as follows:

APP: 5′-GACTGACCACTCGACCAGGTTCTG-3′
  5′-CTTGTAAGTTGGATTCTCATATCCG-3′
  PS1: 5′-AATAGAACGGCAGGAGCA-3′
  5′-GCCA-TGAGGGCACTAATCAT-3′
  GLT-1: 5′GAGGGAAAGTTTGAGTTACCAGC-3′
  5′-CATGTTCACCCTCACAGCAACT-3′

PCR was conducted under the following conditions:

For APP, 94°C for 3 min, 94°C for 30 s, 60°C for 30 s, and 72°C for 30 s for 30 cycles, followed by extension at 72°C for 10 min.
  For PS1, 94°C for 3 min, 94°C for 40 s, 52°C for 40 s, and 72°C for 1 min for 30 cycles, followed by extension at 72°C for 10 min.
  For GLT-1, 94°C for 5 min, 94°C for 30 s, 58°C for 30 s, and 72°C for 30 s for 30 cycles, followed by extension at 72°C for 7 min.
  The lengths of PCR products were as follows: APP, 344 bp; PS1, 608 bp; GLT-1^+^, 865 bp; GLT-1^–^, 385 bp. Samples were loaded in 2% agarose gels. A gel imaging and analysis system (GENE, United Kingdom) was used for imaging.

### Novel Object Recognition Test

The test was run in four black training boxes (40 cm × 40 cm × 30 cm), which were placed in a sound proof lucifuge cabinet. The whole experiment was recorded by a video camera above the chamber. The data were collected and analyzed using the DigBehv Animal Behavior Analysis Software (Shanghai Jiliang Software Technology Co., Ltd., China). The test lasted for 5 days and was divided into habituated stage, acquisition stage, and retention stage, as reported previously (Blurton-Jones et al., 2009). The habituated stage lasted for 3 days; mice were placed in a box, without any objects, for 5 min/day, so they could adapt to the environment. In the acquisition stage (on day 4), mice were exposed to two wooden cubes (3 cm × 3 cm × 3 cm) for 3 min. The cubes were fixed to the floor of the box, 8 cm from the wall, and object location was counterbalanced during the behavior test. The mice were placed in the training box with their noses equidistant from the two objects. In the retention stage (on day 5), one of the cubes was randomly replaced with a hemisphere (4 cm in diameter), which was made of the same material and was as attractive as the cube. The mice were allowed to explore the two objects freely for 3 min. The exploratory behavior of the mice was as follows: the tip of the nose of the mice touched the object or was within 2 cm of the object. The recognition ability of the mice to new objects was evaluated using the recognition index, which was calculated as: time spent on novel object/(time spent on novel object + time spent on familiar object) × 100%. After each trial, the floor of the box and the objects were wiped with 70% ethanol to dispel any preference due to smell.

### Morris Water Maze Test

The test was conducted in a sound proof room in which several visual cues were set in fixed positions. The plastic tank was 120 cm in diameter and 50 cm high, filled with water, in which the temperature was maintained at 20 ± 1°C during the experiment. An escape platform with a diameter of 9 cm was placed in the first quadrant, 1.0 cm below the water surface. A video camera and DigBehv Animal Behavior Analysis Software (Shanghai Jiliang Software Technology Co., Ltd., China) were used for tracking the search bias of mice and analyzed their behavioral performance. Space navigation and retention tests were performed. The navigation test lasted for 4 days with five trials per day. At every trial, mice were put in water, using one of the four start points randomly, with their nose toward the wall and allowed to swim and search the platform. If the mice failed to find the platform successfully in 60 s, the mice were guided to the platform and allowed to remain there for 15 s. The time that the mouse took to climb onto the platform within 60 s and stay there for more than 3 s was determined as escape latency. The average escape latency of five trials was used for statistical analysis. The retention tests were conducted on the fifth navigation of the fourth day. After removing the platform, mice were put in the water from the contralateral quadrant of the platform and allowed to swim for 60 s. The time spent in the target quadrant and the times crossing the platform site were determined as reflection of the retention performance.

### Western Blot Analysis for GLT-1 and xCT Expression

The mice were decapitated under anesthesia. The hippocampus was dissected out and homogenized in 10 volumes of lysis buffer, contained protease inhibitors (Roche, Germany). The homogenates were centrifuged at 12,000 rpm/min for 15 min at 4°C and supernatants were analyzed. The concentration of protein of the supernatants was determined using BCA assay kit (Solarbio, Beijing, China). Thereafter, samples containing protein of 30 μg were loaded on 12% SDS-polyacrylamide gel and then transferred onto polyvinylidene difluoride membranes (Millipore, Billerica, MA, United States) at 21 V for 1 h. Thereafter, the membranes were blocked in 5% bovine serum albumin (BioFroxx, Germany) at 37°C for 1 h, and incubated at 4°C for 12 h with primary antibodies against GLT-1 (polyclonal antibody derived from guinea pig, 1:2,000, Cat. No.: AB1783, Lot No.: 2987435, Millipore, United States), xCT (monoclonal antibody derived from rabbit, 1:2,000, Cat. No.: ab175186, Lot: GR3235736-1, Abcam, United States), and β-actin (monoclonal antibody derived from rabbit, 1:10,000, Cat. No.: AC026, Lot: 9100026001, ABclonal, United Kingdom). Subsequently, the membranes were washed with TBST and incubated at 37°C for 1 h with secondary antibodies for GLT-1 (anti-guinea pig IgG labeled with biotin derived from goat, 1:3,000, Cat. No.: 16-17-06, Lot No.: 130670, KPL, United States), and xCT and β-actin (HRP-labeled anti-rabbit IgG derived from goat, 1:2,000, Cat. No.: 074-1506, Lot No.: 140740, KPL, United States). For GLT-1, an additional incubation with streptavidin conjugated with HRP (1:2,000, Cat. No.: 43-4323, Lot No.: 1513798A, Invitrogen, United States) was conducted at 37°C for 1 h. All membranes were visualized using an ECL reagent (Vazyme, Nanjing, China) after TBST washes. The integral optical density (IOD) of the band was measured with an analysis tool (Alpha Imager, United States). The ratio of IOD of the target protein band to the β-actin band was calculated as the relative change in expression of target protein.

### Immunohistochemistry for GLT-1 Expression

The mice were anesthetized with isoflurane, and perfused via the ascending aorta with normal saline and 4% paraformaldehyde. The brain was removed, and a coronal slice of 3 -mm thickness, including the bilateral hippocampus, was cut. After fixation with 4% paraformaldehyde overnight, the brain slices were embedded in paraffin. Sections of 4-μm thickness were cut. After deparaffinization with xylene and hydration in a descending series of alcohol, sections were incubated with hydrogen peroxide (3%, Lot: AH07183973, Bioss, Beijing, China) for 20 min to remove endogenous peroxidase, heated in a microware in citrate buffer (0.01 M, pH = 6.0) for 18 min for antigen repairing, and then blocked in 10% goat serum (Lot: AH07183973, Bioss, Beijing, China) for 40 min at 37°C. Thereafter, the sections were incubated with primary antibodies against GLT-1 (the same as that used for Western blot, 1:500) overnight at 4°C. After PBS washes, the sections were incubated with secondary antibody (anti-guinea pig IgG labeled with biotin derived from goat, Lot No.: AH07183973, Bioss, Beijing, China) for 1 h at 37°C. Then sections were washed with PBS and incubated with streptavidin working solution conjugated with horseradish peroxidase (Lot: AH07183973, Bioss, China) for 1 h at 37°C. Detections were done using DAB kit (Lot: K166622C, Zhongshan, China). The integral optical density (IOD) of immunostaining was analyzed by Image J to determine the relative expression of GLT-1.

### Glutamate Uptake

The GLT-1 uptake capacity was assayed with ^3^H-glutamate, as described previously (Hu et al., 2015). Briefly, the hippocampus of the mouse was rapidly separated and cut into a small piece in trypsin (0.25%) at 37°C. After the digestion by trypsin for 10 min, Hank’s solution containing calcium and magnesium was added to the samples to block the digestion. The samples were blown 40–60 times in Hank’s solution and then centrifuged at 1,000 rpm/min for 10 min at 4°C. After discarding the supernatant, the cell pellets were resuspended in 500 μl of Hank’s solution for GLT-1 uptake assay. The samples containing 50 μg of protein, determined by the BCA protein assay kit, were incubated with ^3^H-glutamate solution (100 μl, 50 nmol/L) at 37°C for 15 min, in the absence (total glutamate uptake) or presence (non-specific glutamate uptake) of dihydrokainate (0.18 mmol/L, Lot: 064M4608V, Sigma, United States), the specific inhibitor of GLT-1. An ice bath was used to terminate the reaction. The cell suspensions were centrifuged at 1,000 rpm/min for 10 min, and the supernatant was discarded. Thereafter, 1 ml of Hank’s solution was added to the tube, and the cell suspensions were centrifuged again at 1,000 rpm/min for 10 min, with the supernatant being discarded. The cell pellets were lysed using NaOH (100 μl, 3 M) for 20 min. Thereafter, the lysed cell solution was transferred to Whatman filter. After drying at 60°C for 30 min, the filters were placed in a sample bottle (PerkinElmer, United States) containing 2 ml of scintillation liquid. Counts per minute (cpm) were determined by a liquid scintillation counter. The specific glutamate uptake capacity of GLT-1 was calculated through subtracting non-specific glutamate uptake from total uptake.

### Glutathione Assay

The glutathione levels in the hippocampus were measured by fluorometry (Naletova et al., 2018; Piao et al., 2018) using an assay kit (Fluorometric, ab65322, Lot: GR3254829-6, Abcam, United States), according to protocols described in the instruction. The hippocampus of each animal was homogenized in 10 volumes of cell lysis buffer, on an ice bath, and then centrifuged at 12,000 rpm/min for 10 min at 4°C. The supernatant was collected and deproteinized as indicated in the protocol of the assay kit. The standard solution (100 μl) and sample (40 μl samples + 60 μl cell lysis buffer) were incubated with 2 μl of GST reagent and 2 μl of MCB for 5 min. Thereafter, the optical density of fluorescence was immediately measured at Ex/Em = 360/460 nm in the kinetic mode. The concentration of glutathione was expressed as μg/ml.

### Statistics

SPSS 21 was used for statistical analysis. All the data were presented as mean ± standard deviation (SD). The escape latency in Morris water maze test was analyzed using repeated measures ANOVA. Other data were compared through one-way ANOVA combined with Tukey *post hoc* test. A value of *p* < 0.05 was considered statistically different.

## Results

### GLT-1 Knockdown Inhibited the Improvement of Cef on Cognitive Deficit in APP/PS1 Mice

**FIGURE 2:**
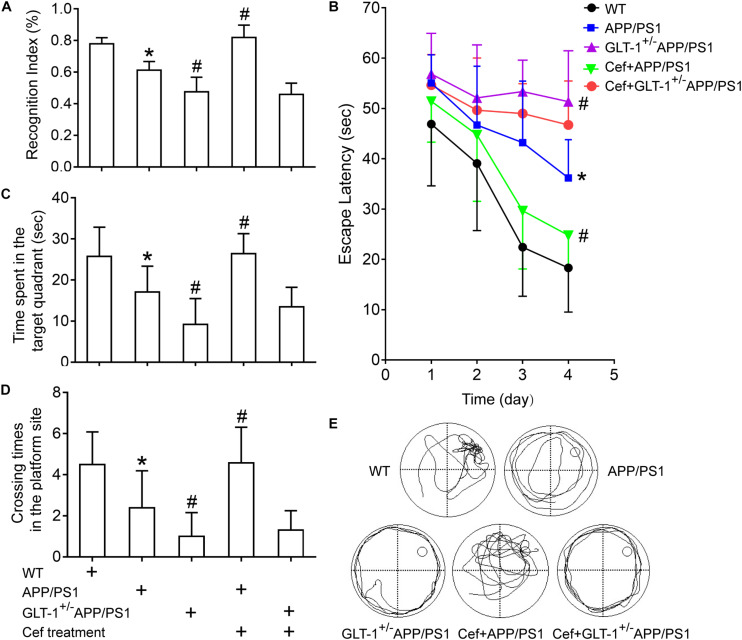
The effect of GLT-1 knockdown on the cognitive improvement induced by Cef in 6 month-old APP/PS1 mice tested by novel object recognition **(A)** and Morris water maze tests **(B–E)**. *p < 0.05 vs. the wild-type group, #*p* < 0.05 vs. the APP/PS1 group. All the data were presented as mean ± standard deviation (SD) and the number of animals in each group in each test is shown in Table 1. Cef treatment increased the cognitive index in novel object recognition test **(A)**, decreased the escape latency **(B)**, and increased the time spent in the target quadrant and the times crossing the platform site **(C–E)** in Morris water maze test in the Cef + APP/PS1 group in comparison with the APP/PS1 group, while little effect in the Cef + GLT-1^±^APP/PS1 group compared with the GLT-1^±^APP/PS1 group was seen.

The novel object recognition tests indicated that [one-way ANOVA, *F*(4,34) = 43.23, *p* < 0.05], in comparison with wild-type mice, the cognitive index in APP/PS1 group significantly decreased (*p* < 0.001). Knockdown of GLT-1 in APP/PS1 mice (GLT-1^±^APP/PS1 mice) further deteriorated the cognitive index compared with APP/PS1 mice (*p* < 0.001). Cef administration obviously increased the cognitive index in Cef + APP/PS1 mice compared with APP/PS1 mice (*p* < 0.001), whereas no changes were seen in Cef + GLT-1^±^APP/PS1 mice compared with GLT-1^±^APP/PS1 mice (*p* = 0.660) (Figure 2A).

Morris water maze tests revealed that [repeated measures ANOVA, *F*(3,12) = 41.1, *p* < 0.05], in comparison with wild-type mice, APP/PS1 mice exhibited longer escape latency in navigation trials (*p* < 0.001). GLT-1 knockdown further increased the escape latency of the APP/PS1 mice in the GLT-1^±^APP/PS1 group in comparison with the APP/PS1 group (*p* = 0.002). During the retention test, APP/PS1 mice exhibited deteriorated retention performances represented with decreases in the time spent in the target quadrant (APP/PS1, *p* = 0.001) and times crossing the platform site (APP/PS1, *p* = 0.002) in comparison with the wild-type group. The GLT-1 knockdown in APP/PS1 mice has deteriorated more the time spent in the target quadrant (*p* = 0.003) and the times crossing the platform site (*p* = 0.034) than those in the APP/PS1 mice. Cef treatment significantly improved the performance in Morris water maze test, including escape latency (*p* = 0.002), the time spent in the target quadrant (*p* < 0.001), and the times crossing the platform site (*p* = 0.001) in the Cef + APP/PS1 mice compared with those in the APP/PS1 mice, while little change was seen in the Cef + GLT-1^±^APP/PS1 mice compared with the GLT-1^±^APP/PS1 mice (escape latency, *p* = 0.171, time spent in the target quadrant *p* = 0.114, times of crossing the platform site *p* = 0.660) (Figures 2B–E).

Thus, novel object recognition and Morris water maze tests indicated that GLT-1 knockdown inhibited the improvement of Cef on cognitive impairment in APP/PS1 AD mice.

### GLT-1 Knockdown Suppressed Cef-Induced Upregulation of GLT-1 Expression and Uptake Activity in APP/PS1 Mice

**FIGURE 3:**
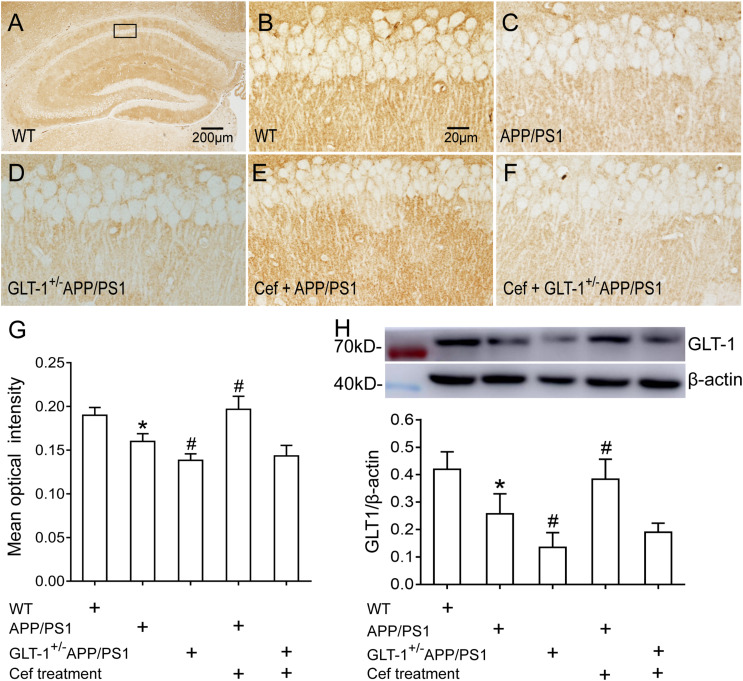
The impact of GLT-1 knockdown on Cef-induced upregulation of GLT-1 expression in the hippocampus of 6-month-old APP/PS1 mouse evaluated by immunohistochemistry and Western blot. **(A–F)** Representative photomicrographs of immunohistochemical staining for GLT-1. The scale bar is 200 μm in **(A)**, and 20 μm in **(B)**, which is available for **(B–F)**. **(G)** A quantitative presentation of immunostaining by mean optical density. **(H)** Western blot analysis for GLT-1 expression, in which the top shows immunoreactive bands of GLT-1 and β-actin, the bottom bar graph is a quantitative presentation of the immunoreactive bands by integral optical density. *p < 0.05 vs. the wild-type group, #*p* < 0.05 vs. APP/PS1 group, *n* = 6 per group. All the data were presented as mean ± standard deviation (SD). Cef treatment upregulated GLT-1 expression in the Cef + APP/PS1 group compared with the APP/PS1 group, but had little effect in the Cef + GLT-1^±^APP/PS1 group in comparison with the GLT-1^±^APP/PS1 group.

Immunohistochemical staining showed that [one-way ANOVA, *F*(4,25) = 35.989, *p* < 0.05], in wild-type mice, a large number of GLT-1 immunoreactive particles (brown) were distributed extensively in the entire hippocampus. APP/PS1 mice exhibited decreased GLT-1 immunostaining in the hippocampus, manifested with uneven distribution and flake deletions of the immunoparticles, and decreased IOD, in comparison with the wild-type group (*p* = 0.001). Knockdown of GLT-1 in the APP/PS1 mice (GLT-1 ± APP/PS1 group) further reduced GLT-1 expression compared with the APP/PS1 mice (*p* = 0.015). Cef treatment of the Cef + APP/PS1 group significantly increased the immunoreactivity of GLT-1 compared with the APP/PS1 group, as seen with more intense immunostaining and increased IOD of the immunoparticles in the hippocampus of the mice (*p* < 0.001 vs. APP/PS1 group). However, Cef treatment of the Cef + GLT-1^±^APP/PS1 group did not increase the immunoreactivity of GLT-1 in comparison with the GLT-1^±^APP/PS1 group (*p* = 0.929) (Figures 3A–G). Western blot assay [one-way ANOVA, *F*(4,25) = 23.995, *p* < 0.05] revealed similar changes as the immunohistochemistry assay on the impact of Cef on GLT-1 expression in APP/PS1 mice without or with knockdown of GLT-1 (Figure 3H).

**FIGURE 4:**
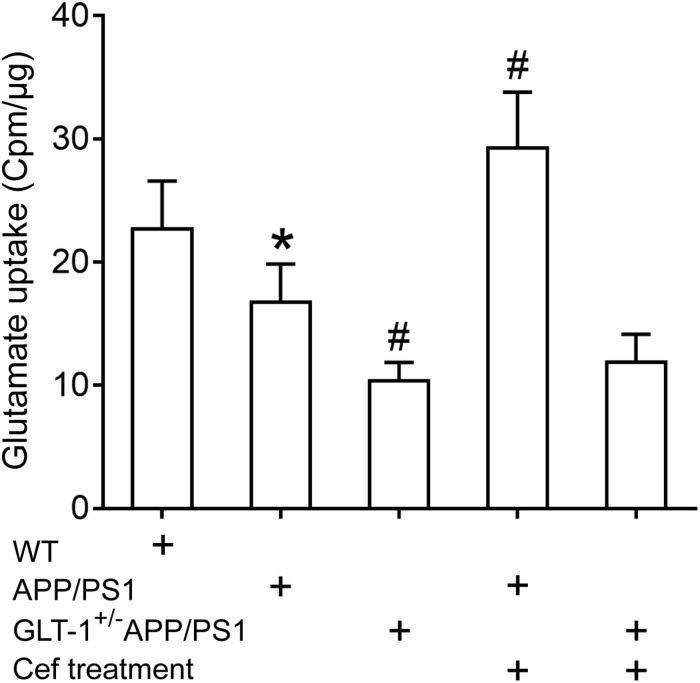
The impact of Cef treatment on glutamate uptake of GLT-1 in the hippocampus and the impact of GLT-1 knockdown on the Cef-induced change in the uptake determined with ^3^H-glutamate. *p < 0.05 vs. wild-type group, #*p* < 0.05 vs. APP/PS1 group, *n* = 6 per group. All the data were presented as mean ± standard deviation (SD). Glutamate uptake of GLT-1 reduced in APP/PS1 mice, and the knockdown of GLT-1 further exacerbated the decrease in the GLT-1^±^APP/PS1 group. Cef treatment restored the decline in APP/PS1 mice, but not in GLT-1^±^APP/PS1 mice.

The GLT-1 uptake assay showed that [one-way ANOVA, *F*(4,25) = 34.931, *p* < 0.05], in comparison with the wild-type group, the GLT-1 uptake capacity was significantly declined in the APP/PS1 group (*p* = 0.031), and the declines were more obvious in the GLT-1^±^APP/PS1 group (*p* = 0.017 vs. APP/PS1 group). Cef treatment obviously reversed the decline in glutamate uptake by GLT-1 in the Cef + APP/PS1 group in comparison with the APP/PS1 group (*p* < 0.001), while there were no obvious changes in the Cef + GLT-1^±^APP/PS1 group in comparison with the GLT-1^±^APP/PS1 group (*p* = 0.927) (Figure 4).

The results revealed that GLT-1 knockdown inhibited Cef-induced upregulation of GLT-1 expression and uptake capacity in APP/PS1 mice.

### The Impact of Cef on xCT Expression and Glutathione Level in APP/PS1 and GLT-1^±^ APP/PS1 AD Mice

**FIGURE 5:**
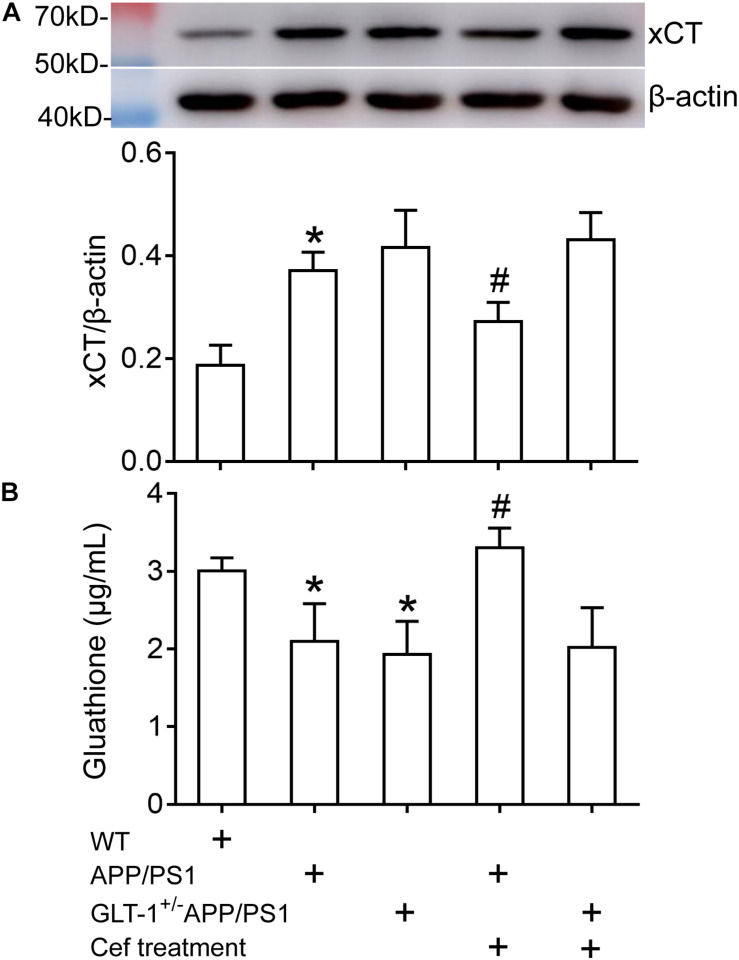
The impact of Cef treatment on xCT expression and glutathione level in the hippocampus of APP/PS1 mice and GLT-1^±^APP/PS1 mice assayed with Western blot analysis and glutathione assay kit. **(A)** Western blot analysis for xCT expression in which the top is the immunoreactive band of xCT and β-actin, the bottom bar graph is quantitative presentation of the immunoreactive bands by integral optical density. **(B)** Glutathione levels. *p < 0.05 vs. the wild-type group, #*p* < 0.05 vs. the APP/PS1 group, *n* = 6 per group. All the data were presented as mean ± standard deviation (SD). xCT expression increased, while glutathione level decreased in APP/PS1 and GLT-1^±^APP/PS1 groups compared with the wild-type group. Cef treatment restored xCT expression and glutathione level in the APP/PS1 group, but had no effect in the GLT-1^±^APP/PS1 group.

Western blot assay showed that [one-way ANOVA, *F*(4,25) = 26.638, *p* < 0.05] there was an obvious upregulation in xCT expression in APP/PS1 mice (*p* < 0.001) in comparison with the wild-type mice. GLT-1 knockdown in APP/PS1 mice (GLT-1^±^APP/PS1 group) seemed to further increase xCT expression, although it was not statistically different in comparison to the APP/PS1 mice (*p* = 0.523). Cef treatment in APP/PS1 mice (Cef + APP/PS1 group) significantly restored xCT expression in comparison with the APP/PS1 group (*p* = 0.013). However, GLT-1 knockdown in APP/PS1 mice (GLT-1^±^APP/PS1 group) prevented Cef from restoring xCT expression level (*p* = 0.984 vs. Cef + GLT-1^±^APP/PS1 group) (Figure 5A).

Glutathione assay showed that [one-way ANOVA, *F*(4,25) = 15.442, *p* < 0.05] the glutathione level in the hippocampus of APP/PS1 mice and GLT-1 knockdown APP/PS1 mice (GLT-1^±^APP/PS1 group) significantly declined in comparison to the wild-type group (*p* = 0.005 in APP/PS1 group and *p* = 0.001 in GLT-1 ± APP/PS1 group). Cef treatment in APP/PS1 mice (Cef + APP/PS1 group) increased glutathione level (*p* < 0.001 vs. APP/PS1 group). However, Cef could not increase glutathione levels in the Cef + GLT-1^±^APP/PS1 group, in comparison with the GLT-1^±^APP/PS1 group (*p* = 0.995) (Figure 5B).

## Discussion

Glutamate transporter-1 is an important molecule in regulating neurotransmission mediated by glutamate, maintaining the appropriate concentration of glutamate and limiting excitotoxic effect of glutamate and neurodegeneration. GLT-1 loss or dysfunction has been implicated in the pathogenesis of AD. For example, postmortem investigation of patients with AD showed that GLT-1 was expressed more in activated astrocytes, and cognitive functions were preserved better before the patient’s death (Kobayashi et al., 2018). Restoring GLT-1 expression by transgenic or pharmacological approaches in experimental animals significantly improved synaptic damage, Aβ deposition, and cognitive impairments (Takahashi et al., 2015; Mi et al., 2018). This suggests that upregulation of GLT-1 expression and uptake for glutamate may be profitable for preserving cognition in AD patients or animal models. Cef has been revealed to selectively increase GLT-1 expression and uptake activity, and this upregulation has been suggested to be protective in many models of diseases, such as amyotrophic lateral sclerosis (Rothstein et al., 2005) and Parkinson’s disease (Hsu et al., 2015; Hsieh et al., 2017). Furthermore, Cef can ameliorate tau pathological changes and cognitive impairment by promoting the glutamate transporters in 3xTg AD model (Zumkehr et al., 2015) and long-term potentiation impairment and paired-pulse responses in OKA-induced AD model (Hamidi et al., 2019). We have recently showed that Cef can stimulate GLT-1 expression and improve cognitive impairment in APP/PS1 AD mice (Fan et al., 2018). In the present study, we confirmed the upregulation of GLT-1 expression and improvement on cognitive impairments after treatment of Cef in APP/PS1 AD mice. These findings were in accordance with the previous reports and further proved the improving effect of Cef on cognitive impairment in AD mice and the implication of GLT-1 in the process.

Glutamate transporter-1 scavenges glutamate by glutamate uptake. Previous studies have shown that glutamate transporter mRNA splice variants reduce glutamate uptake in AD-related conditions (Scott et al., 2011). Astrocytes derived from the cortex of patients with AD show a decreased glutamate uptake (Liang et al., 2002). Soluble Aβ could reduce glutamate uptake by 70% in crude synaptosomes of the hippocampus (Li et al., 2009). Overexpressions of mutant APP in the brain of transgenic mice reduce glutamate uptake (Li et al., 1997; Masliah et al., 2000). These results suggest that decreased glutamate uptake activity is involved in the pathogenesis of AD and correlates to cognitive decline (Masliah et al., 1996). Therefore, it is necessary to study the changes in GLT-1 uptake to elucidate the protective mechanism of Cef on cognitive deficits of AD. Thus, we measured glutamate uptake capacity with ^3^H-glutamate in the present study. We found that, accompanied with downregulation in GLT-1 expression, glutamate uptake capacity of GLT-1 significantly declined in APP/PS1 AD mice, and Cef treatment clearly inhibited the decline. These results suggested that the decline in GLT-1 uptake activity is involved in the cognitive impairment of AD mice, and Cef may alleviate cognitive impairment by improving the activity of GLT-1 uptake.

However, in contrast to the improvement of Cef on the cognitive impairment of AD models, there were inconsistent reports in different studies related to the roles of Cef on cognition. For example, chronic administration of Cef in rats induced significant memory impairment in the novel object-recognition test (Matos-Ocasio et al., 2014). In addition, Cef disrupted motor skill learning and the functional outcome following focal ischemic cortical lesions (Kim and Jones, 2013). The detrimental effect of Cef in learning and memory might be associated with differences in animal models or administration approaches. It is necessary to design experiments to observe the influence of inhibiting or blocking GLT-1 expression and/or function to prove the effect induced by GLT-1. Previous work from our laboratory has shown that GLT-1 antisense oligodeoxynucleotides or dihydrokainic acid, a specific inhibitor of GLT-1, inhibited the beneficial effects of GLT-1 in global brain ischemic rats (Cui et al., 2015; Hu et al., 2015). Recently, we revealed that dihydrokainic acid inhibited the improvement of Cef on cognitive impairment in APP/PS1 mice (Fan et al., 2018). To more convincingly prove the improvement of Cef on cognitive impairment of APP/PS1 AD model mice and roles of GLT-1 in the improvement, we constructed partial GLT-1 knockout APP/PS1 mice in the present study. This mouse has phenotypes of APP/PS1 mice with downregulated GLT-1 expression. A previous study has reported that this mouse has more deteriorated impairment in cognitive functions and, thus, is a good animal model for the study to prove the involvement of GLT-1 in cognitive deficits of AD (Mookherjee et al., 2011).

Using this mouse in the present study, we showed that GLT-1 protein was significantly decreased, and Cef treatment had little upregulating effect on the downregulated GLT-1 expression in the GLT-1^±^APP/PS1 mice. The results confirmed the effectiveness of the gene modification in knocking down GLT-1 expression. In addition, the knockdown of GLT-1 aggravated the cognitive dysfunction of the APP/PS1 mice in novel objective recognition and Morris water maze tests, which were consistent with a previous report (Mookherjee et al., 2011). The results suggested the involvement of GLT-1 impairment in the pathogenesis of AD. However, we are concerned whether GLT-1 loss inhibits the Cef-induced improvement of cognition. It was clearly shown that the improvement of Cef on cognitive impairment of APP/PS1 mice was inhibited after GLT-1 knockdown, i.e., the administration of Cef had no significant improvement on the cognitive impairment in GLT-1^±^APP/PS1 mice. Simultaneously, the promoting impact of Cef on the activity of GLT-1 uptake in APP/PS1 mice was weakened in GLT-1^±^APP/PS1 mice. The findings provide convincing evidence for the conclusion that Cef improves cognitive impairment of APP/PS1 AD mice by upregulating GLT-1 expression and uptake activity. Although it has been reported that GLT-1 knockdown might increase GLAST expression, another glutamate transporter (Pardo et al., 2006), whether other glutamate transporters and their cell type-specific expression, and glutamate receptors, including ionotropic and metabotropic glutamate receptors, are impacted after GLT-1 knockdown, and whether these influences participate in the cognitive improvements after Cef treatment in APP/PS1 AD mouse remain to be clarified. The elucidation of these issues would promote the understanding of the effects of GLT-1 knockdown and ceftriaxone treatment on APP/PS1 AD mouse.

In addition to GLT-1, xCT is functionally related to glutamate homeostasis and GLT-1 uptake activity. Another novelty of the present study is that changes in xCT expression before and after Cef treatment in APP/PS1 and GLT-1^±^APP/PS1 AD mice has been observed. We found that xCT expression was upregulated in APP/PS1 and GLT-1^±^APP/PS1 mice. This finding is supported by previous reports. For instance, xCT expression in the cerebral cortex of adult Aβ PP23 mice increased, accompanied with a decreased glutamate uptake and increased extracellular glutamate (Schallier et al., 2011). Aβ injection increased xCT gene expression in the hippocampus of adult mouse (Qin et al., 2006). Amyloid precursor protein evoked glutamate export from xCT, which led to synaptic degeneration and neuronal death (Barger and Basile, 2001). These results indicated that xCT is involved in the pathological progression of AD. We think that increased xCT expression might be a compensatory change in the impairment of GLT-1 expression and uptake activity in APP/PS1 and GLT-1^±^APP/PS1 mice. In our previous study, we have shown the downregulation of GLT-1 expression in APP/PS1 mice (Fan et al., 2018), and in the present study, we have shown a decline in the uptake activity of GLT-1. As the main function of GLT-1 is the uptake of glutamate, the decrease in GLT-1 expression and uptake activity leads to the accumulation of glutamate and an increase in extracellular glutamate concentration. Since glutamate is a competitive inhibitor of glutamate export of xCT, excess accumulation of glutamate in the extracellular space impedes the xCT activity, leading to decreases in the export of glutamate and then the import of cystine (Murphy et al., 1989; Patel et al., 2004). Consequently, xCT expression increases to compensate the impaired transporting activity and maintain normal transporting activity for glutamate and cystine. It has been reported that xCT expression increases under conditions of glutamate oxidative stress and GSH depletion (Lewerenz et al., 2006, 2013), which support the explanation.

With changes in xCT expression, the glutathione level in APP/PS1 mice decreased in comparison with that in wild-type mice. This decrease might result from oxidative stress induced by the excitotoxicity of glutamate and accumulation of Aβ in AD mice, which consume a lot of glutathione. Another factor causing the decrease in glutathione in AD mice might be associated with dysfunctions in the transporting activity of xCT, resulting from the accumulation of glutamate and increased extracellular glutamate concentration induced by the dysfunction of GLT-1 uptake activity mentioned above. It seems conflicting that xCT expression was upregulated, while glutathione content decreased in APP/PS1 mice. We think that this might result from the incomplete compensatory role of xCT expression and the following transporting activity. It means that the compensatory upregulation of xCT expression could not completely compensate the overdepletion and insufficient synthesis of glutathione resulting from oxidative stress and disturbed xCT transporting activity for cystine, respectively. Notably, Cef treatment restored the glutathione level to about sham level in the present study, and this effect was inhibited by GLT-1 knockdown in APP/PS1 mice, which suggested that Cef can regulate xCT activity by improving GLT-1 uptake activity. This result is consistent with a previous report that Cef can increase glutathione levels by activating transcription factor Nrf2 (Lewerenz et al., 2009) and is particularly valuable to increase the antioxidant capability and beneficial effects in diseases, such as AD.

Several studies reported that Cef increases xCT expression in fibroblasts and hippocampal cell line HT22 (Lewerenz et al., 2009, 2013), and in the nucleus accumbens core in rat modeled reinstatement of cocaine seeking (LaCrosse et al., 2017). In the present study, we found that upregulation of xCT expression in APP/PS1 AD mice was restored after Cef treatment, which means that the xCT expression in Cef-treated APP/PS1 AD mice was downregulated, rather than upregulated, compared with no Cef-treated APP/PS1 AD mice. This result seems in conflict with previous reports mentioned above. We believe that the change in xCT expression after Cef treatment in APP/PS1 AD mice might be, at least partly, a secondary change following GLT-1 upregulation induced by Cef treatment (Supplementary Material). It has been shown that excess glutamate in the extracellular space is a competitive inhibitor of xCT activity (Murphy et al., 1989; Patel et al., 2004). The upregulated GLT-1 expression and glutamate uptake resulting from Cef treatment decreased the extracellular glutamate concentration, which eliminated the inhibition of xCT activity due to the high extracellular glutamate concentration, and thus, xCT expression was restored in the APP/PS1 mice. Notably, that GLT-1 knockdown in APP/PS1 mice prevented Cef from restoring xCT expression and glutathione levels further proved the above assumption. Although we could not show upregulation in xCT expression after Cef treatment in APP/PS1 mice, our data are in agreement with that Cef can play a supporting effect for xCT activity by stimulating GLT-1 expression and uptake for glutamate, which promotes glutamate uptake of astrocytes and then supplies intracellular and reduces extracellular glutamate, respectively. This supporting effect promotes the activity of xCT, drives more cystine import to astrocytes, and increases synthesis of glutathione (Rimaniol et al., 2001; Lewerenz et al., 2006).

## Data Availability Statement

The data that supports the findings of this study are included in the article, further information is available from the corresponding author upon reasonable request.

## Ethics Statement

The animal study was reviewed and approved by the Animal Care and Use Committee of Hebei Medical University.

## Author Contributions

JG conducted most parts of the experiments and wrote the manuscript. LZL, SF, and LRL participated in parts of the experiments. CL and SL supplied the animals of the GLT-1 knockdown. WBL and XHX designed the study, performed the data analysis and interpretation, and reviewed the manuscript. All authors contributed to the article and approved the submitted version.

## Conflict of Interest

The authors declare that the research was conducted in the absence of any commercial or financial relationships that could be construed as a potential conflict of interest.
