# Supplementary material for: GLT-1 Knockdown Inhibits Ceftriaxone-Mediated Improvements on Cognitive Deficits, and GLT-1 and xCT Expression and Activity in APP/PS1 AD Mice
Source: Front Aging Neurosci. 2020 Oct 6;12:580772. doi: 10.3389/fnagi.2020.580772 (PMC7574737; doi:10.3389/fnagi.2020.580772)
Supplement: Supplementary file 1 [file Table_1.DOCX]

Supplementary material : the original gels of western blot


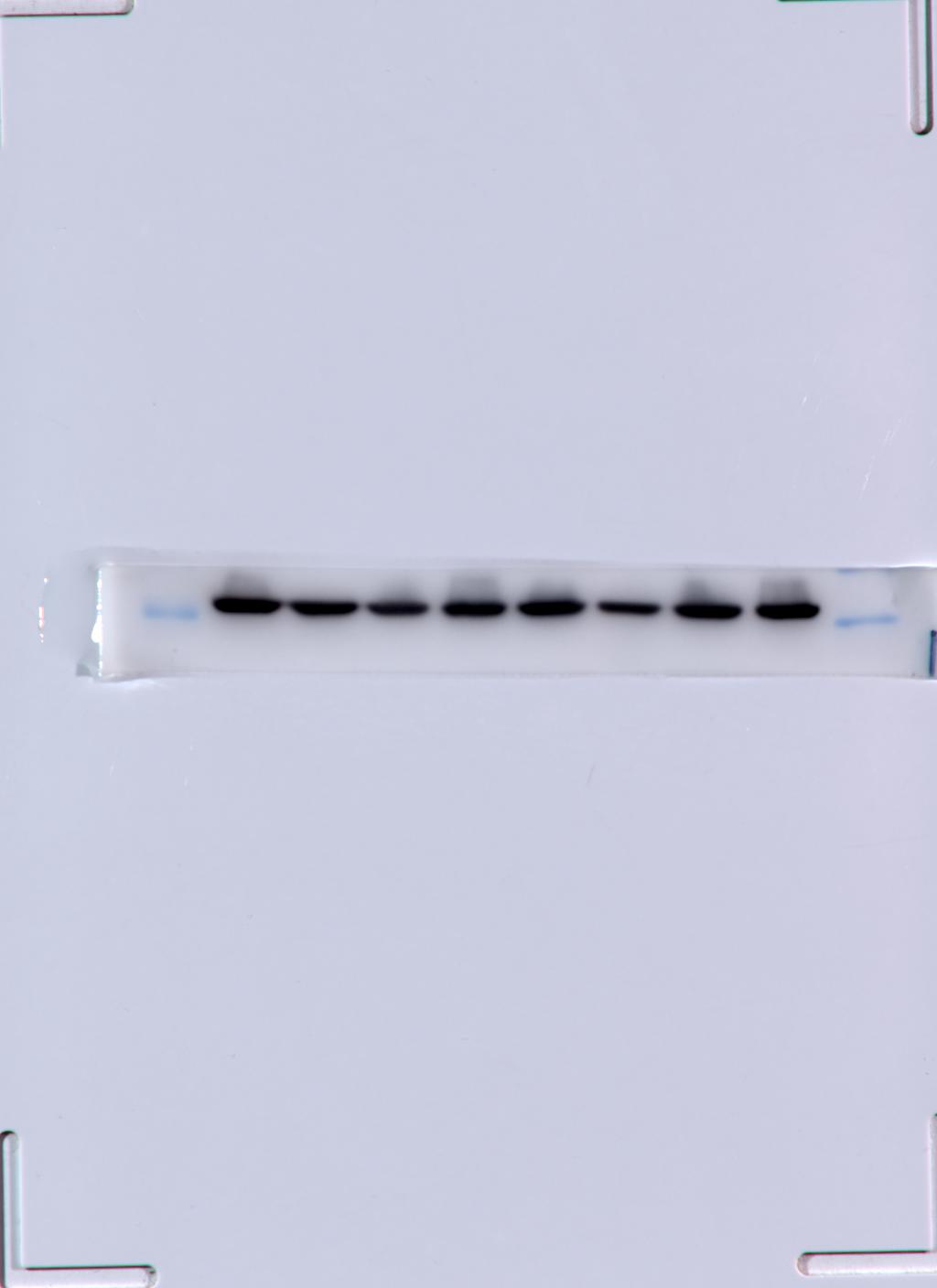

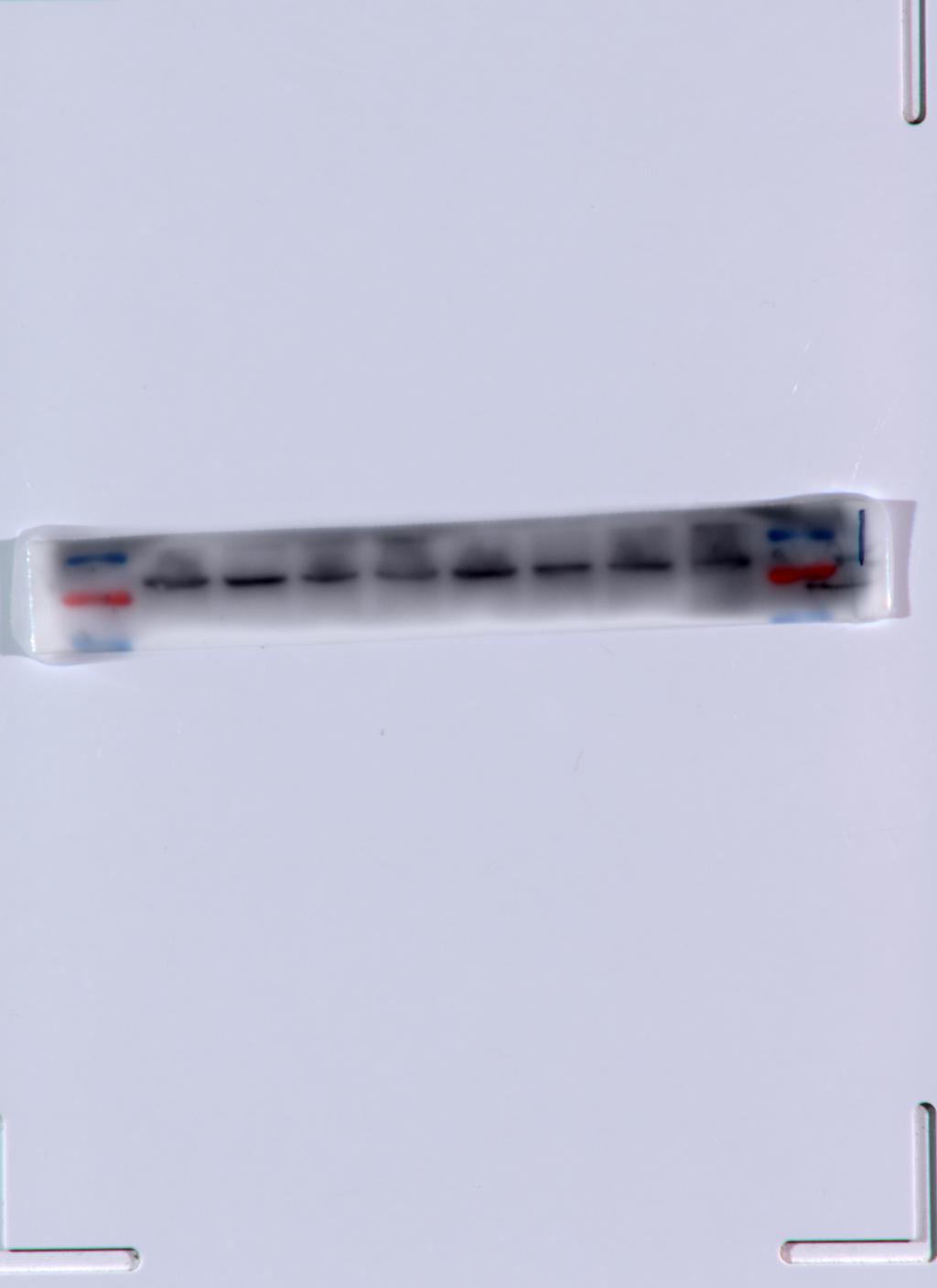


GLT-1

β-actin

GLT-1

β-actin

GLT-1

blot 1 (used in Fig 1C in the

manuscript as representative blot)

blot 2

GLT-1

β-actin


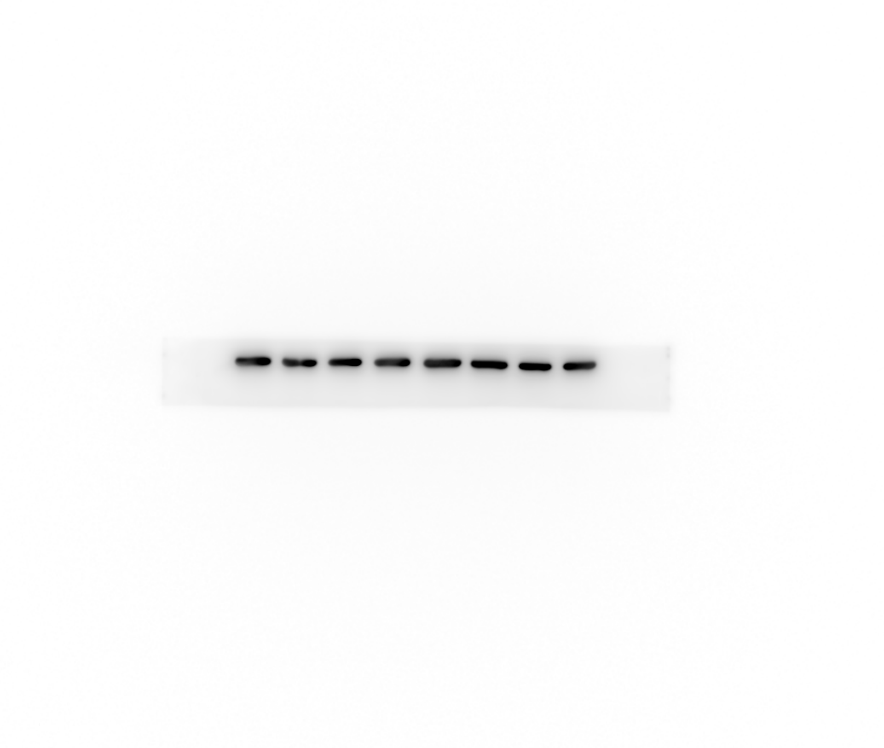

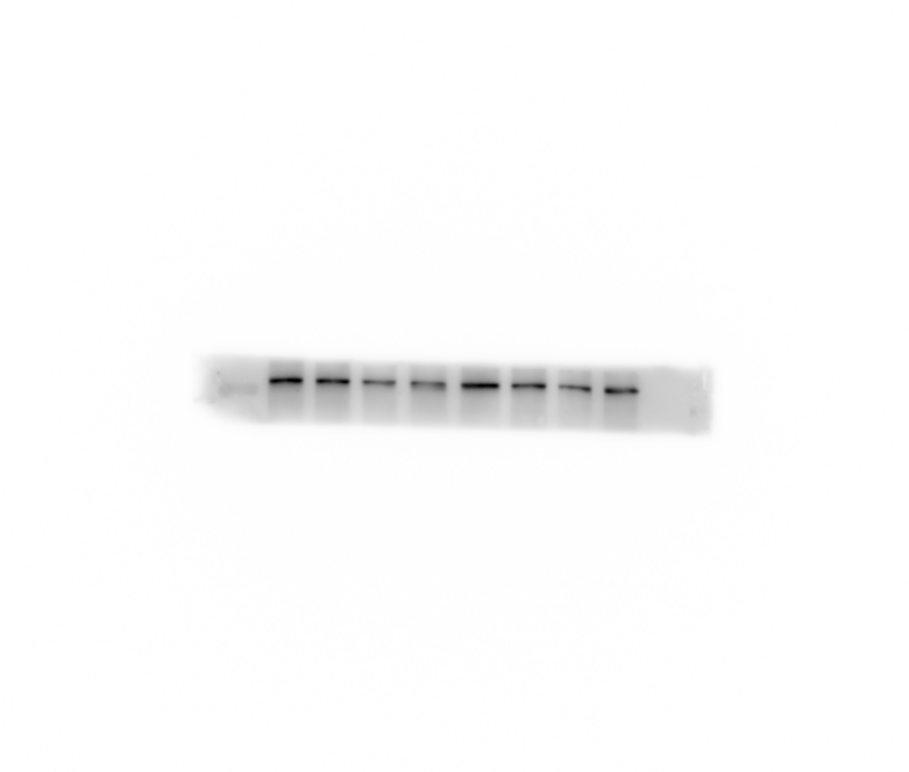

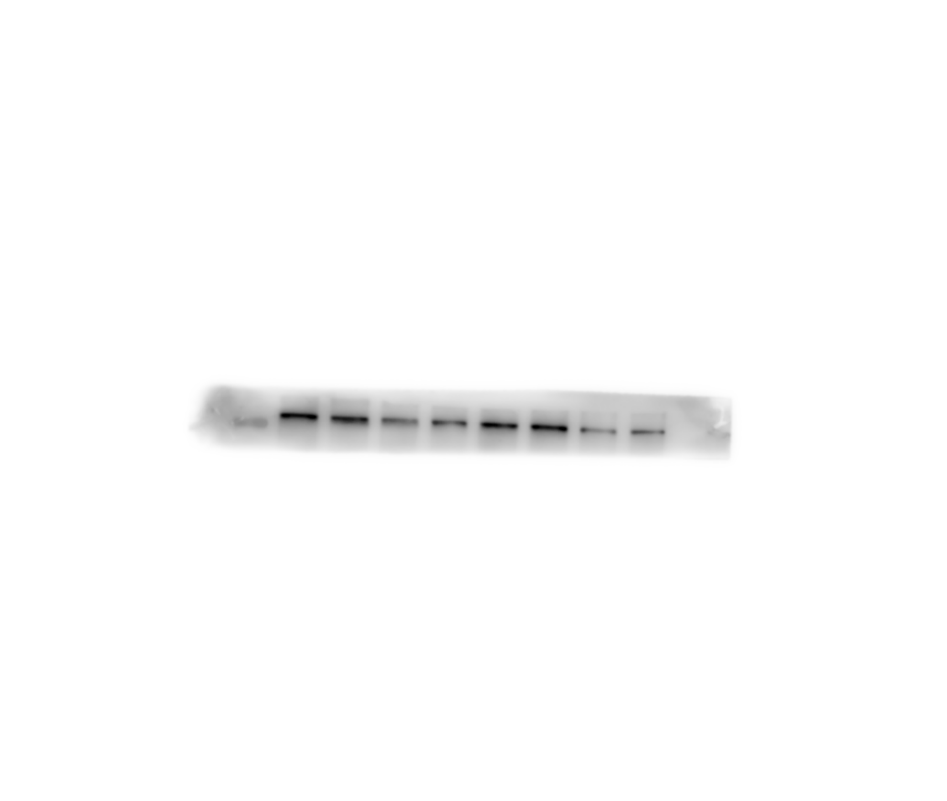

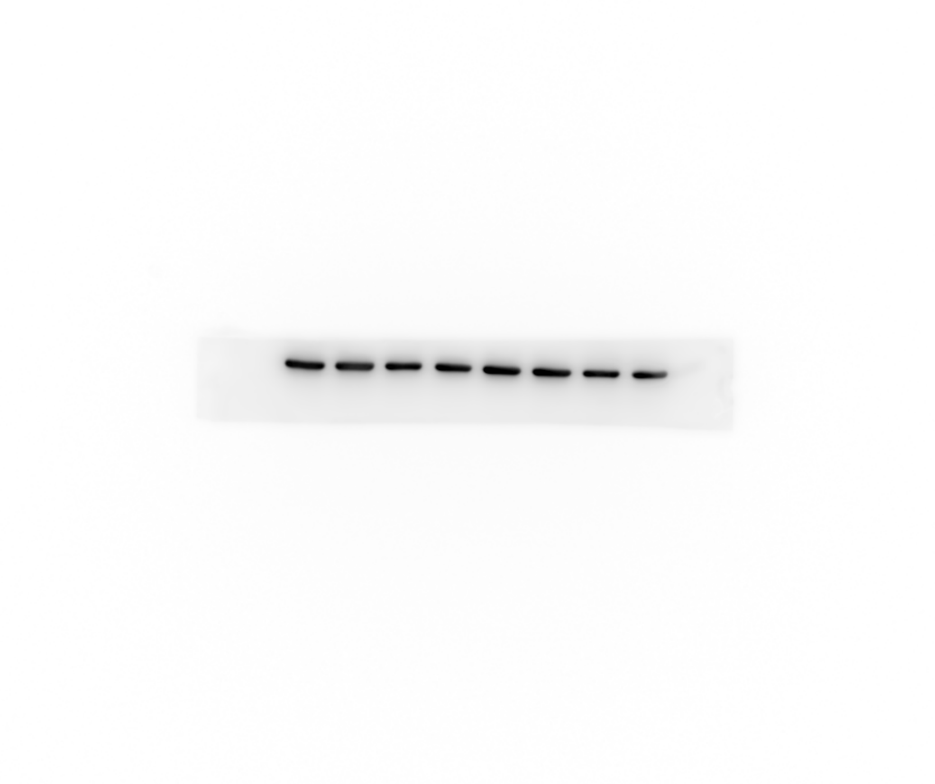


**1. The western blot bands of GLT-1 protein in C57 and GLT-1 knockdown C57 mice**

The lane order from left to right on blot 1-3 is: C57, C57, C57^+/-^, C57^+/-^, C57, C57, C57^+/-^, C57^+/-^

The lane order from left to right on blot 4 is: C57, C57, C57, C57^+/-^, C57^+/-^, C57^+/-^

blot 3

blot 4

β-actin


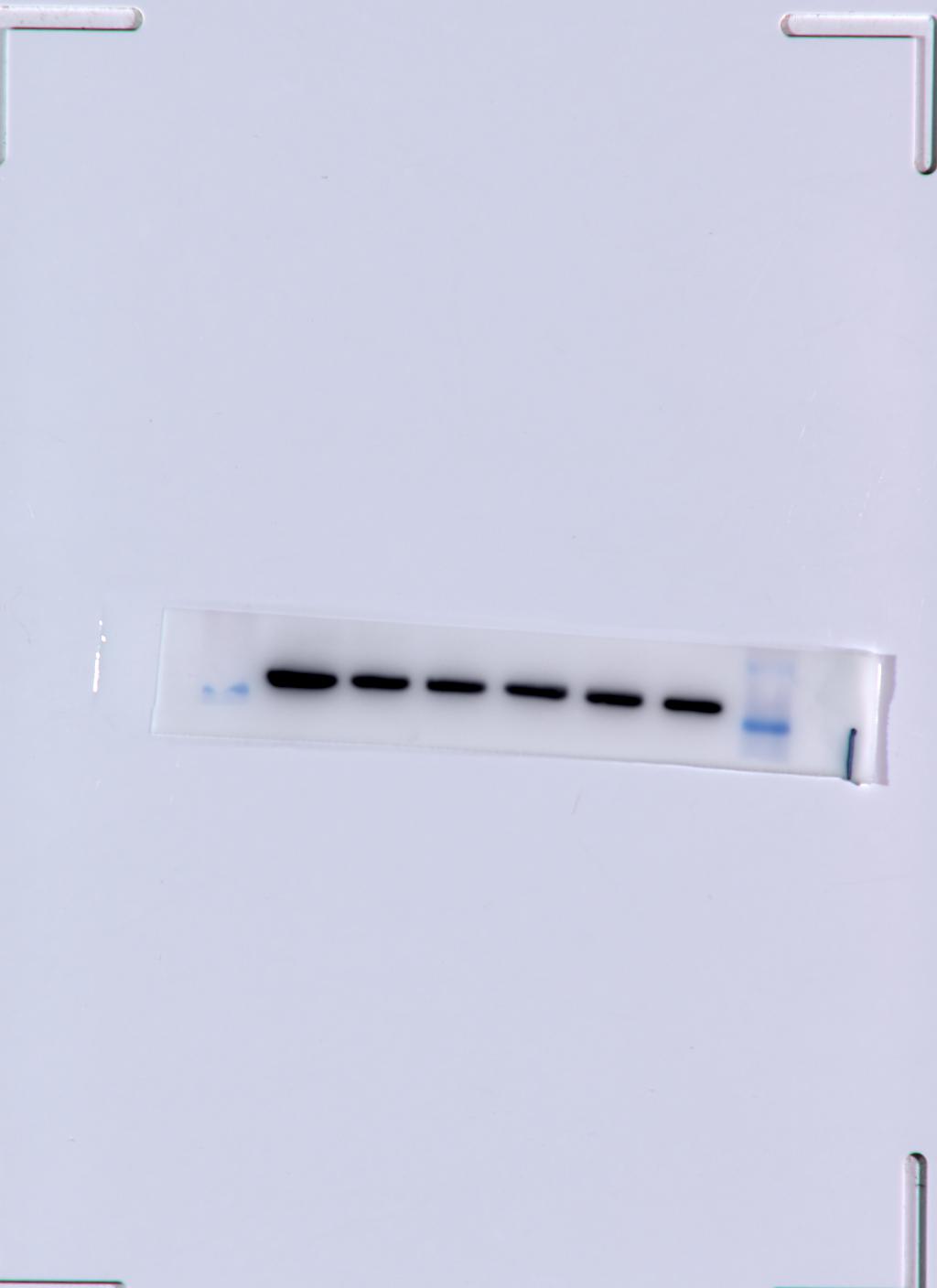

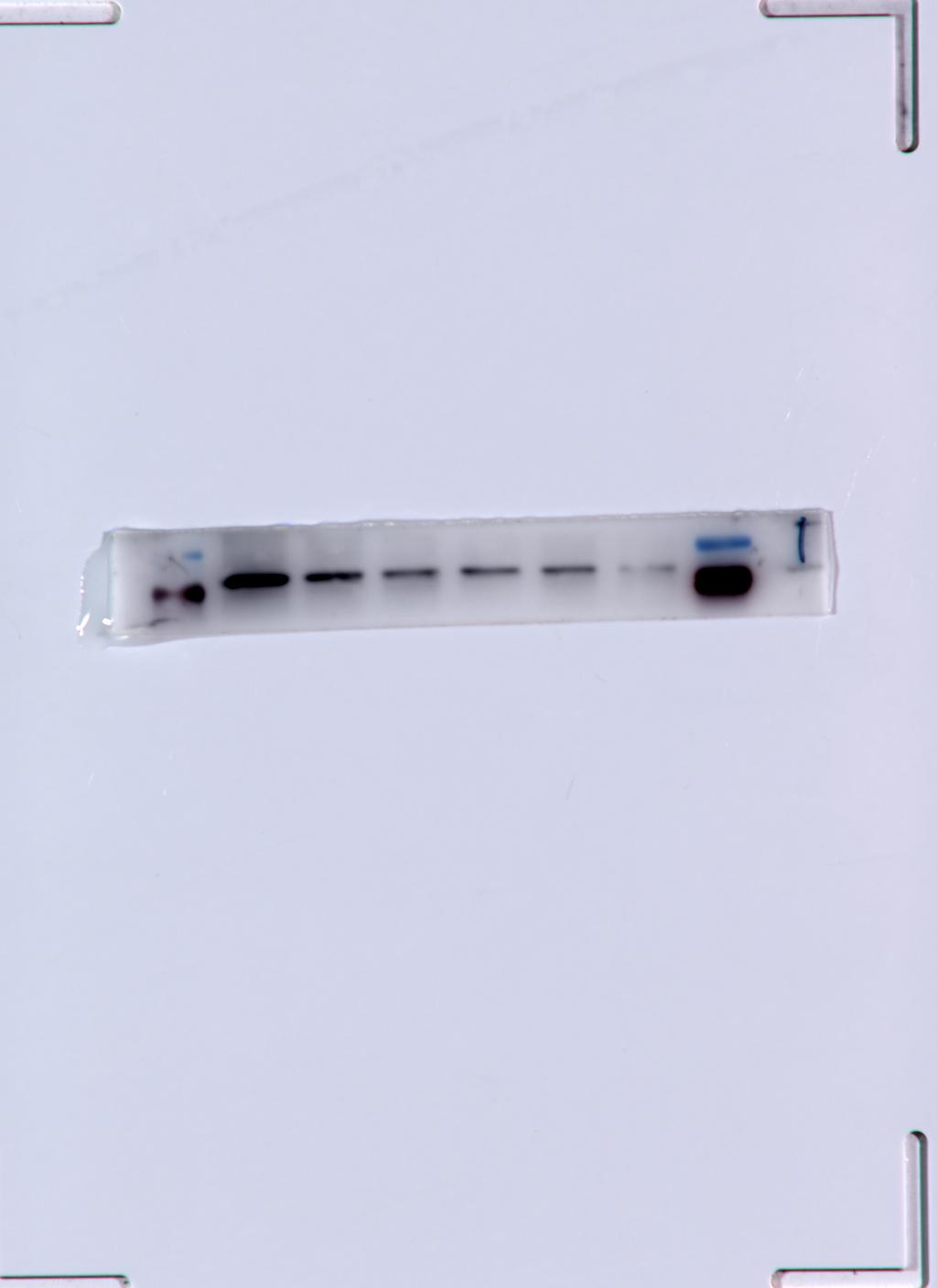


**2. The western blot bands of GLT-1 protein in each group**

The lane order from left to right on blot 1-5 is: C57, APP/PS1, GLT1^+/–^APP/PS1, Cef+APP/PS1, Cef+GLT1^+/–^APP/PS1.

The lane order from left to right on blot 6-7 is: C57, APP/PS1, GLT1^+/–^APP/PS1, Cef+APP/PS1, Cef+GLT1^+/–^APP/PS1, C57, APP/PS1, Cef+APP/PS1, Cef+GLT1^+/–^APP/PS1.


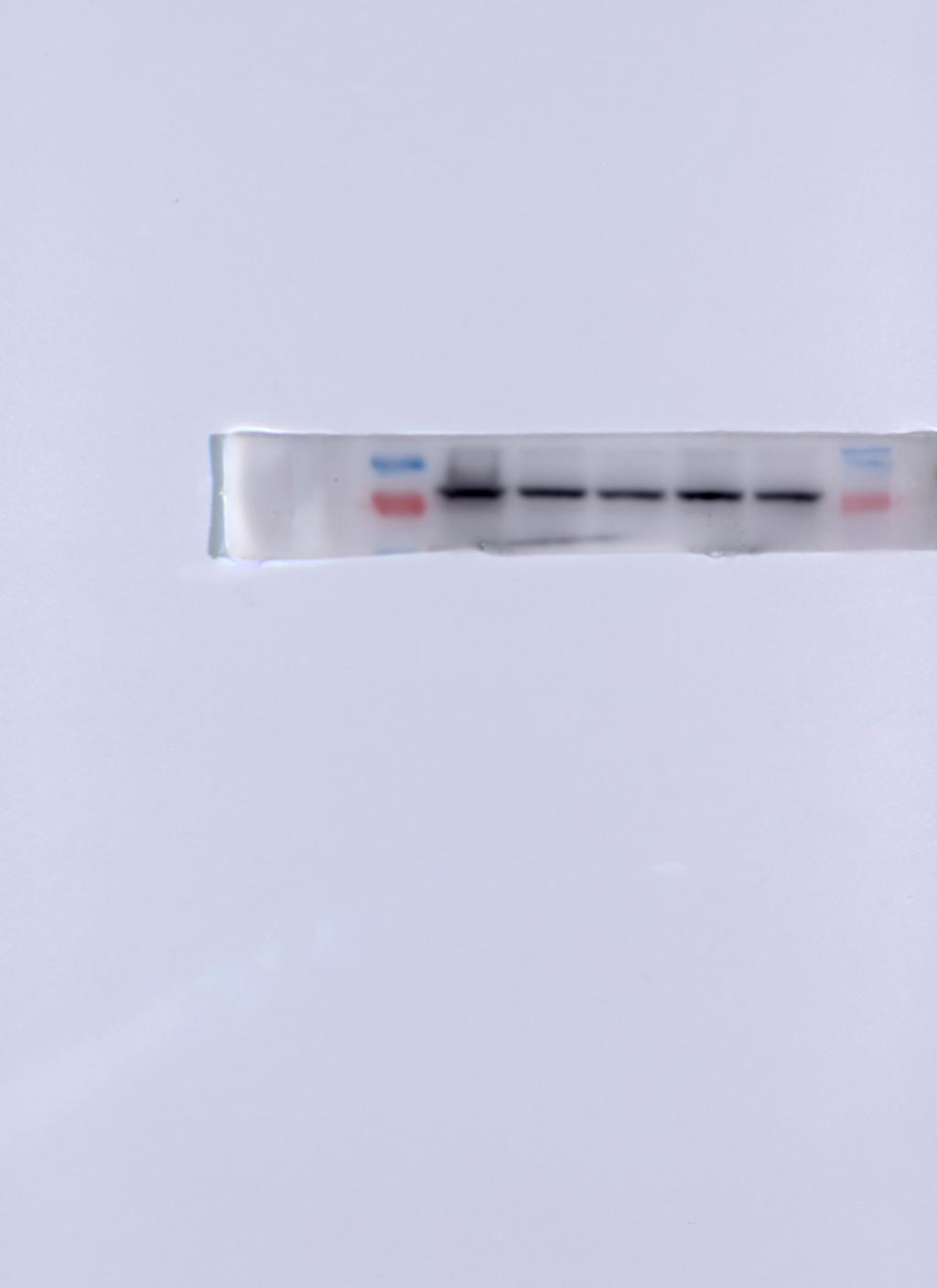

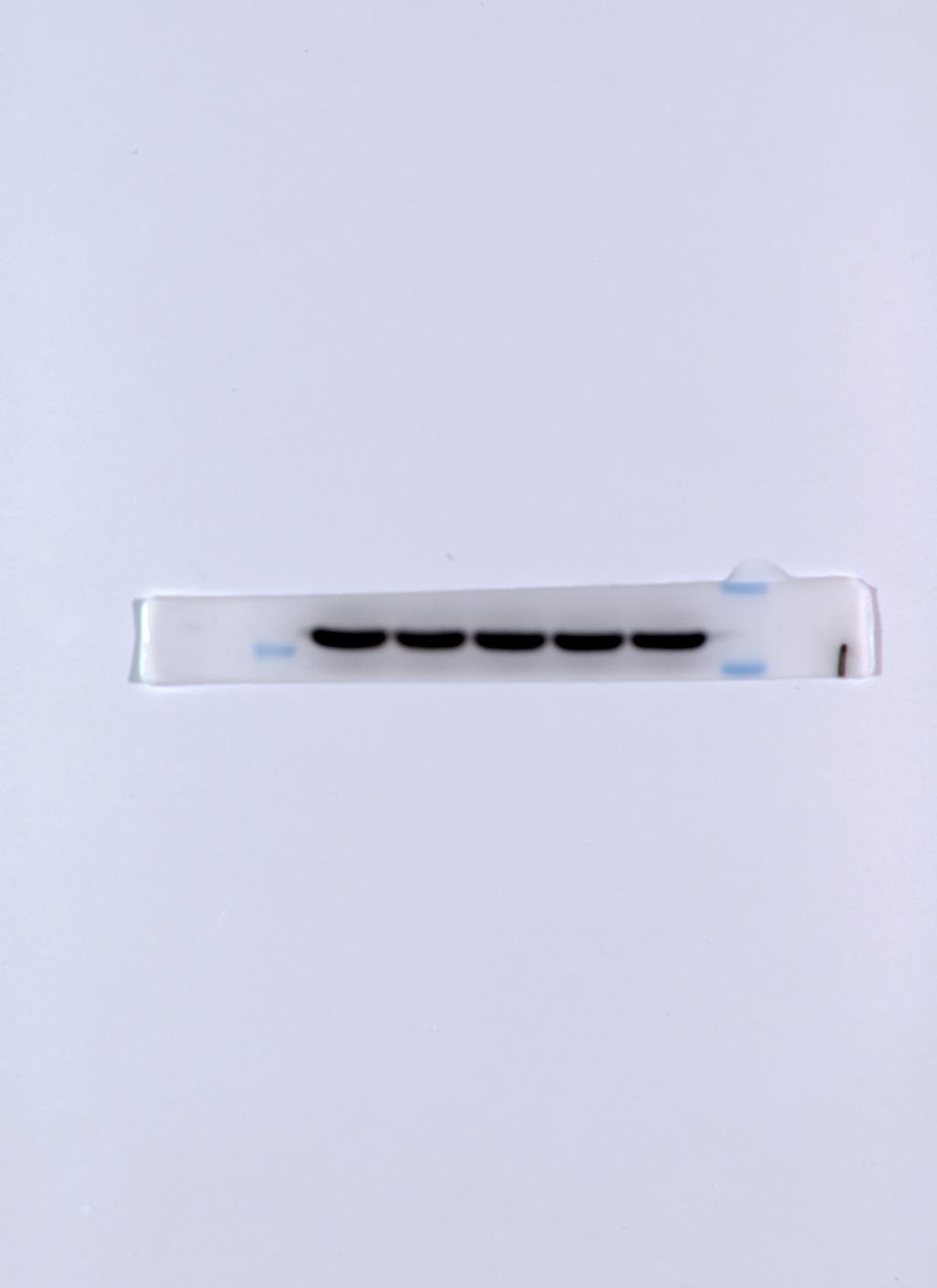


β-actin

GLT-1

β-actin

GLT-1


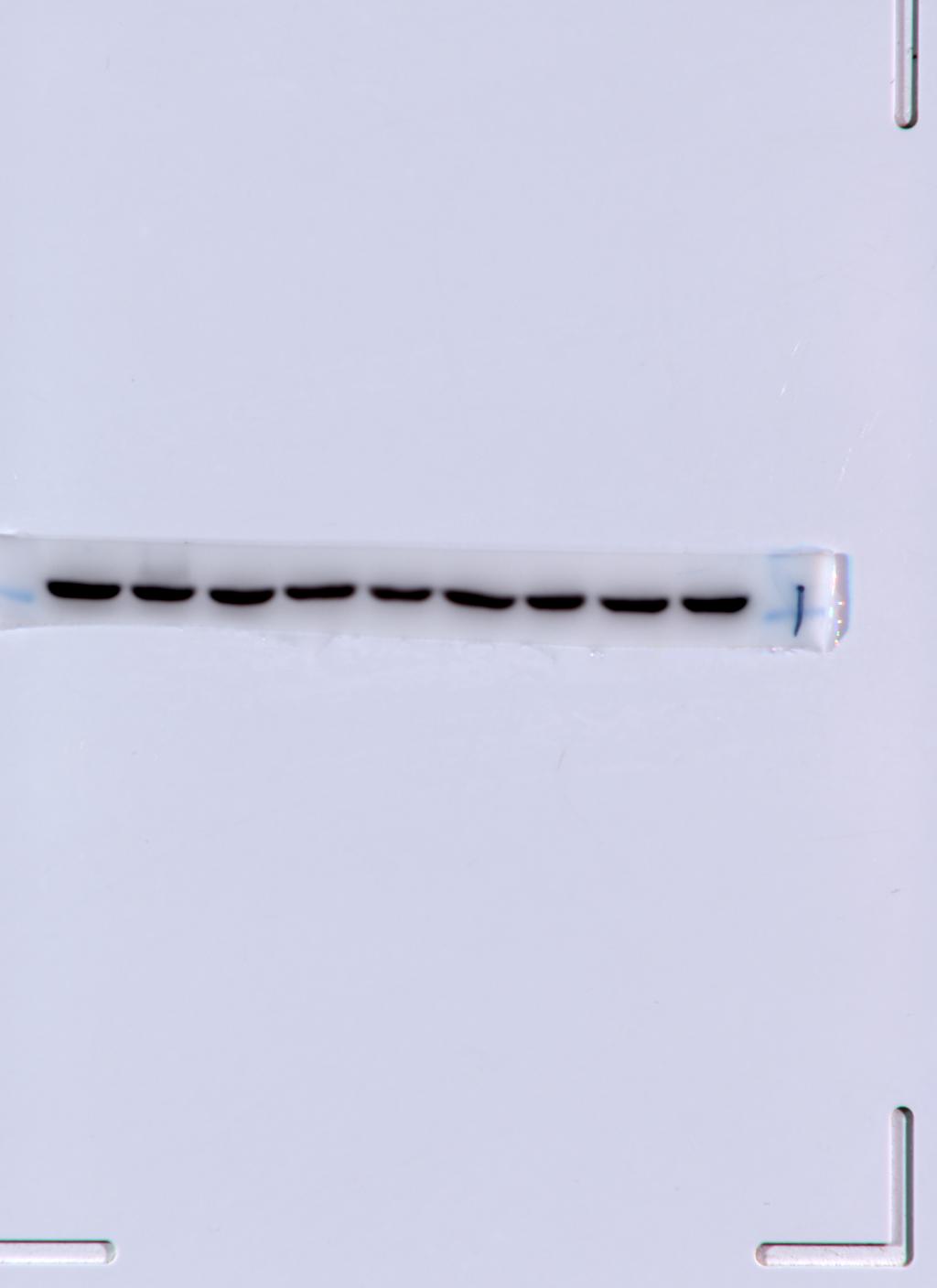

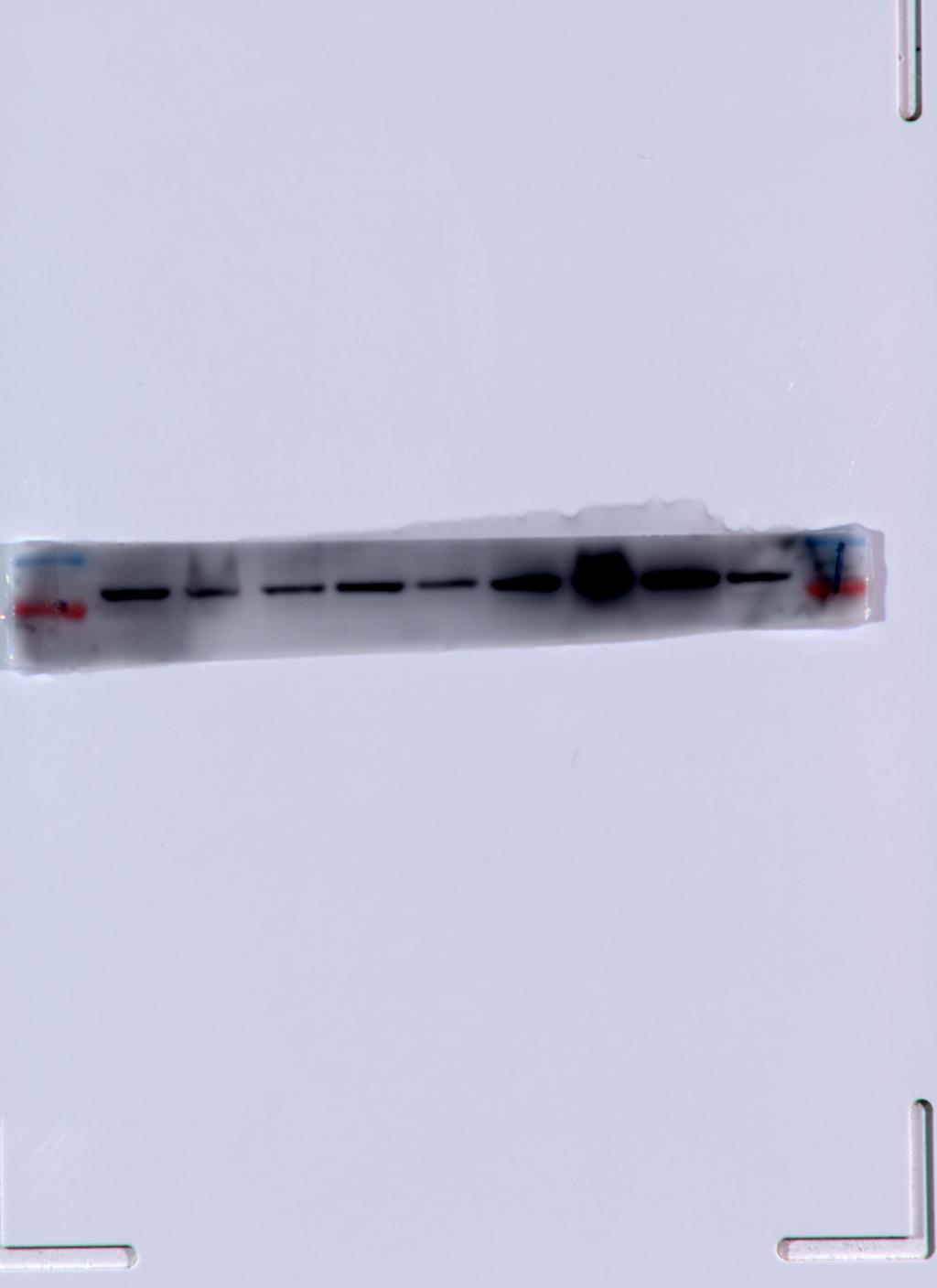

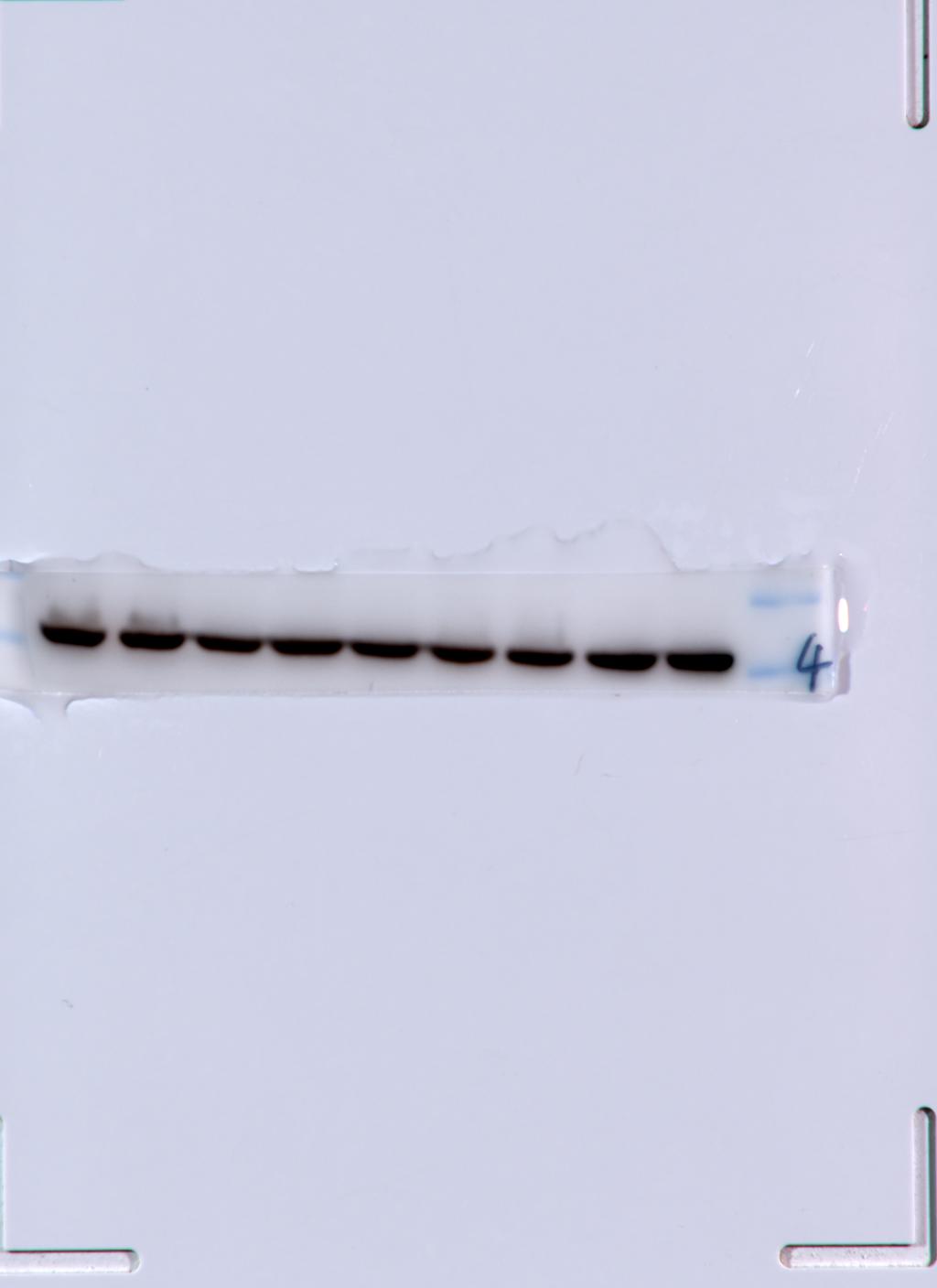

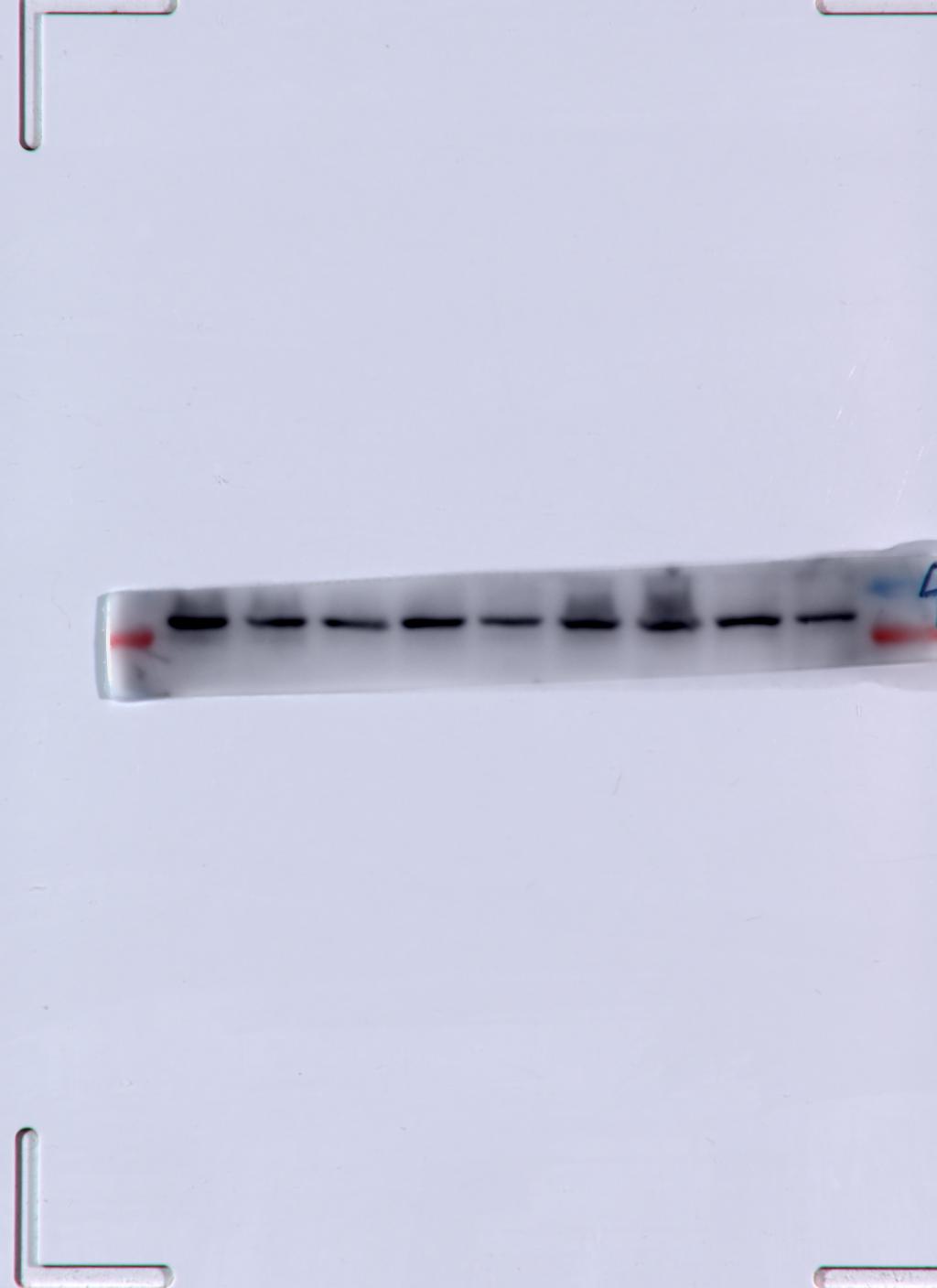


blot 7

blot 6

β-actin

GLT-1

β-actin

GLT-1


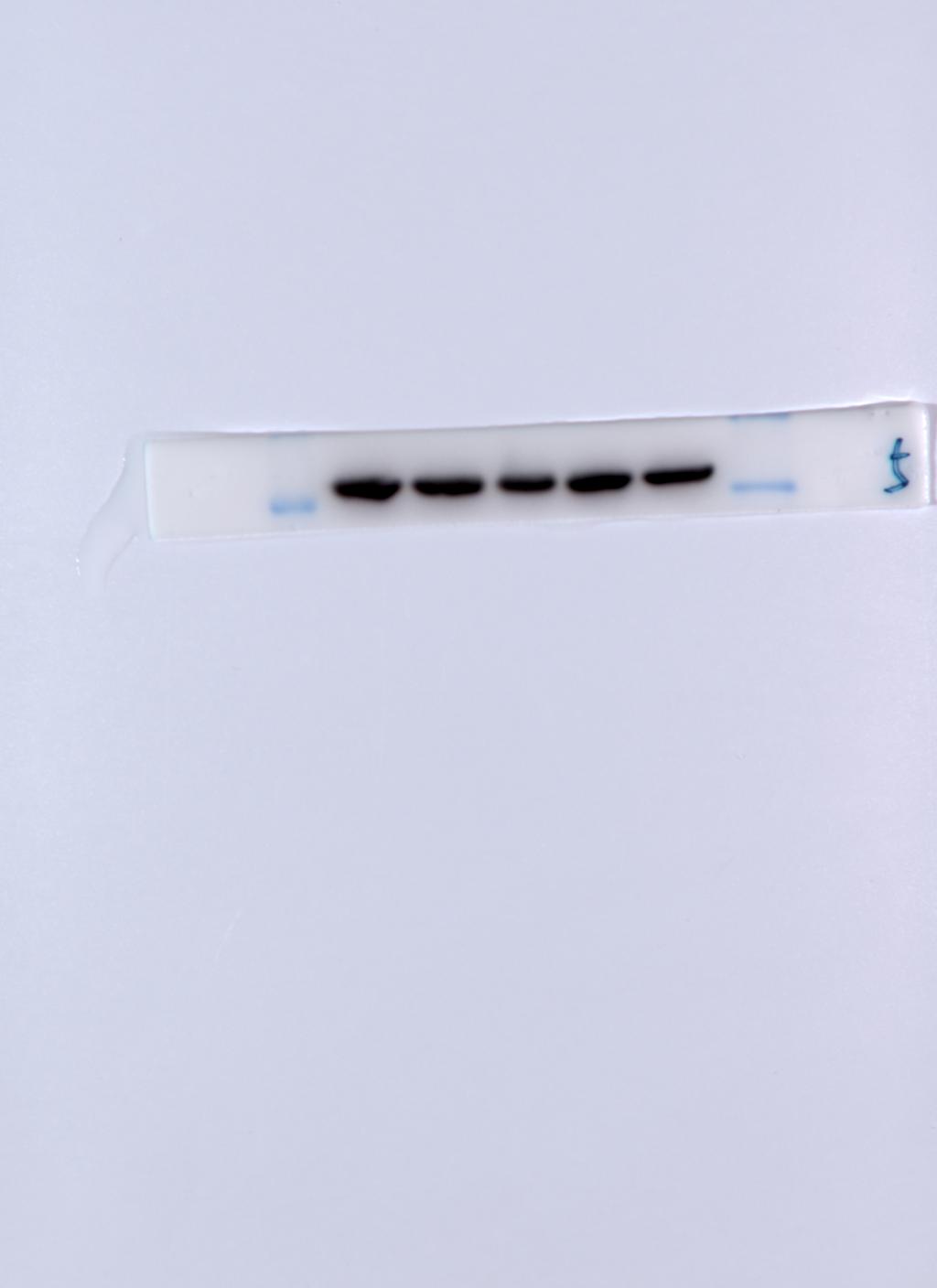

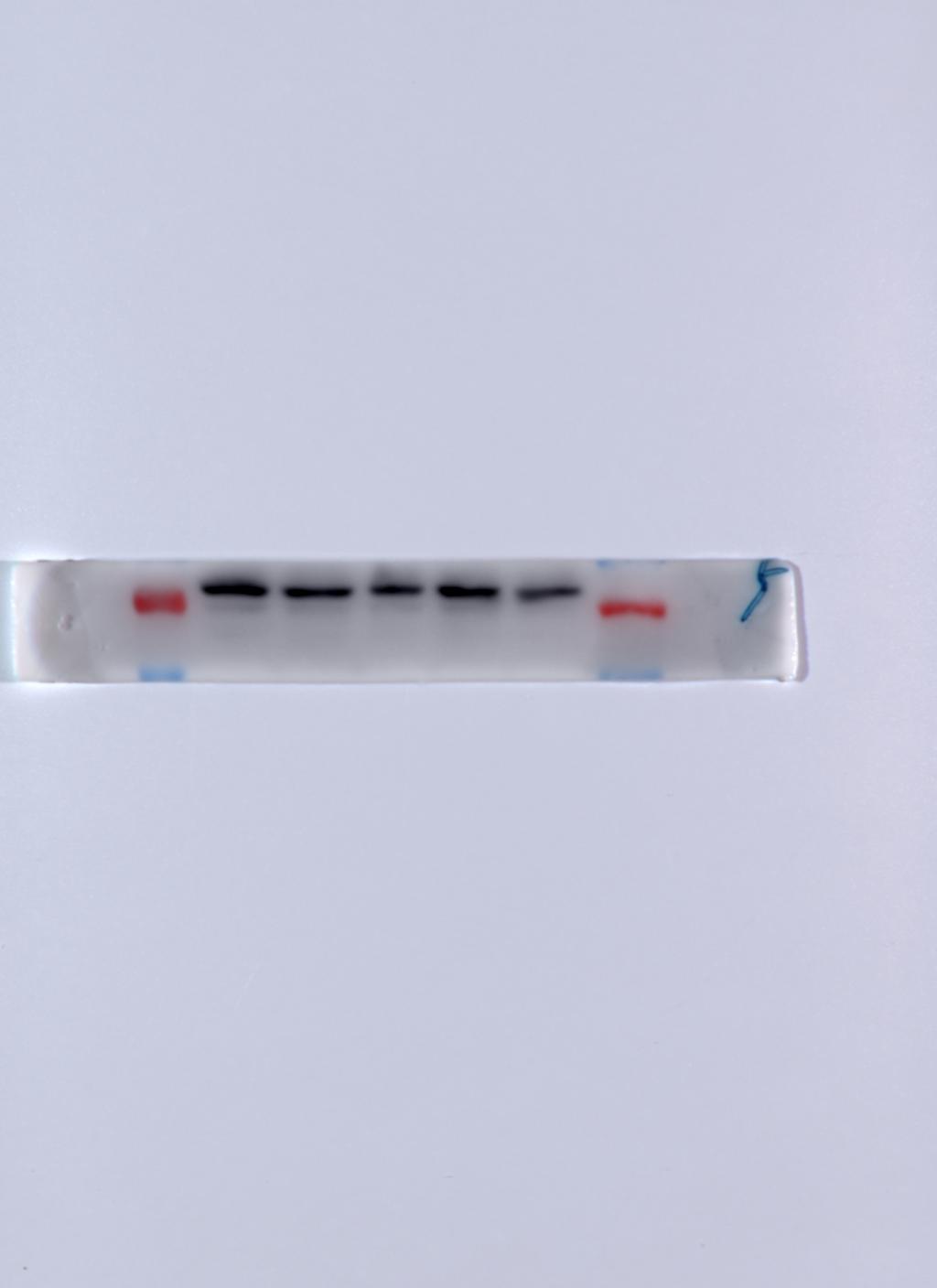


blot 5

blot 4

β-actin

GLT-1

β-actin

GLT-1


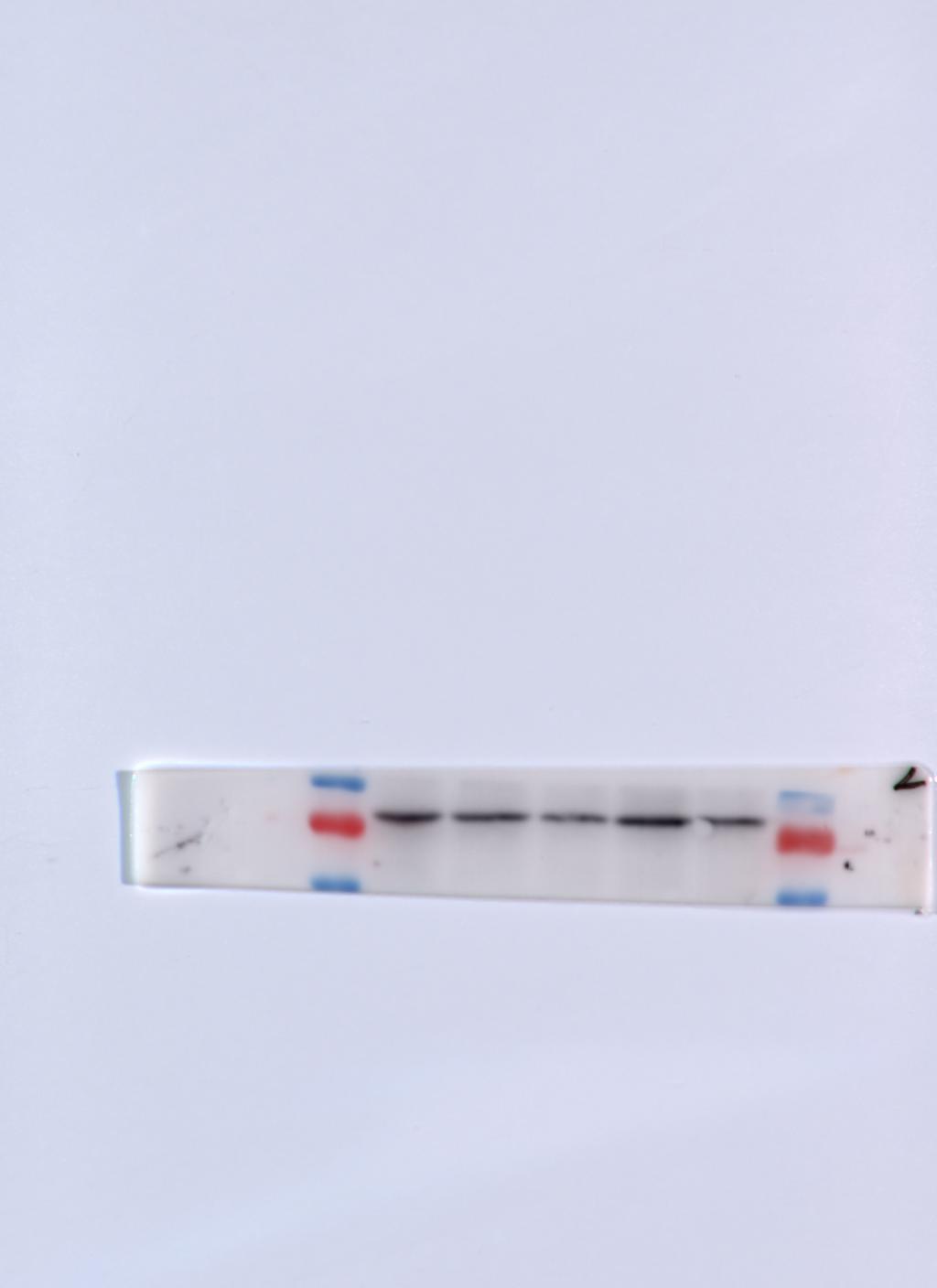

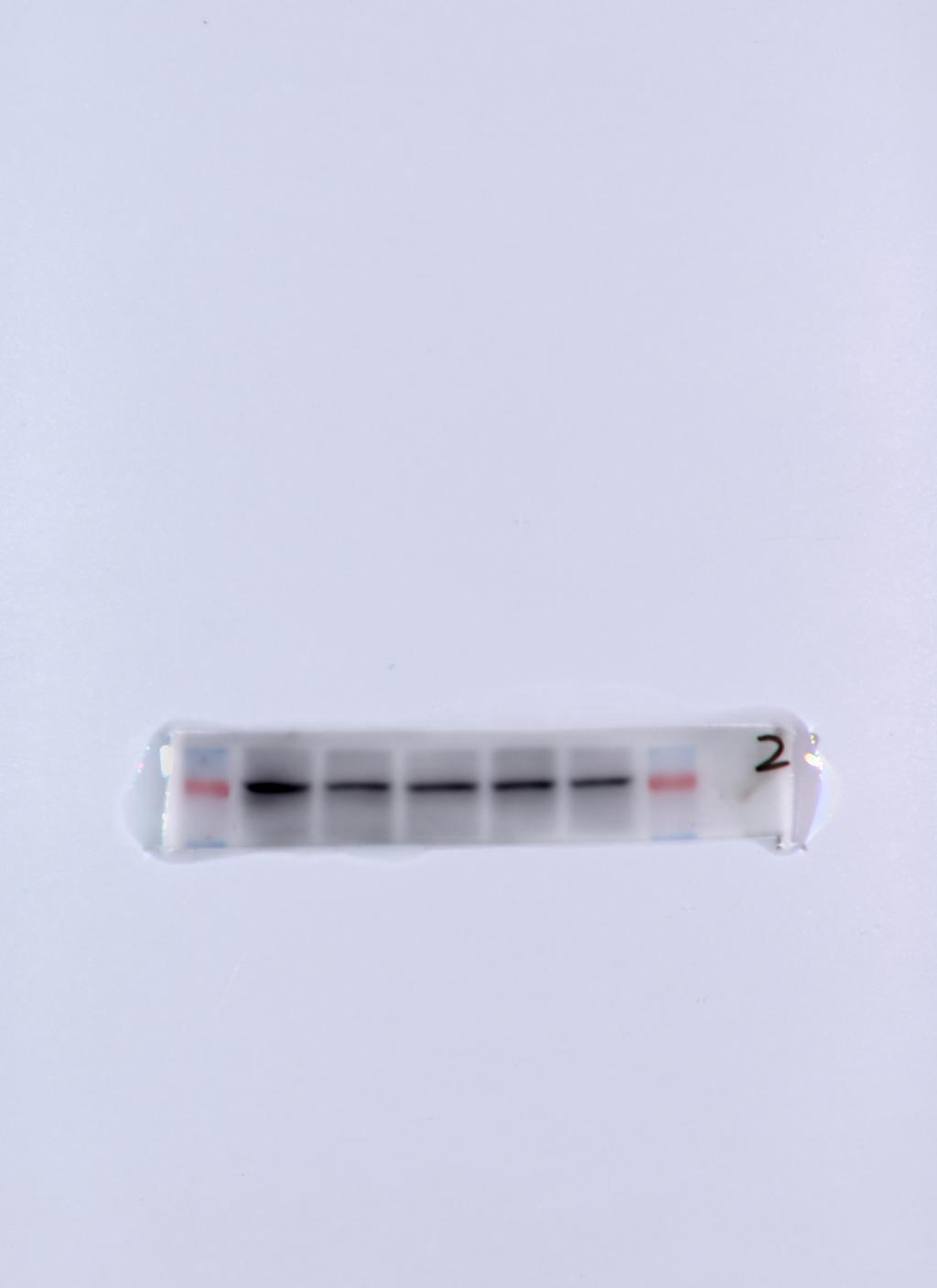

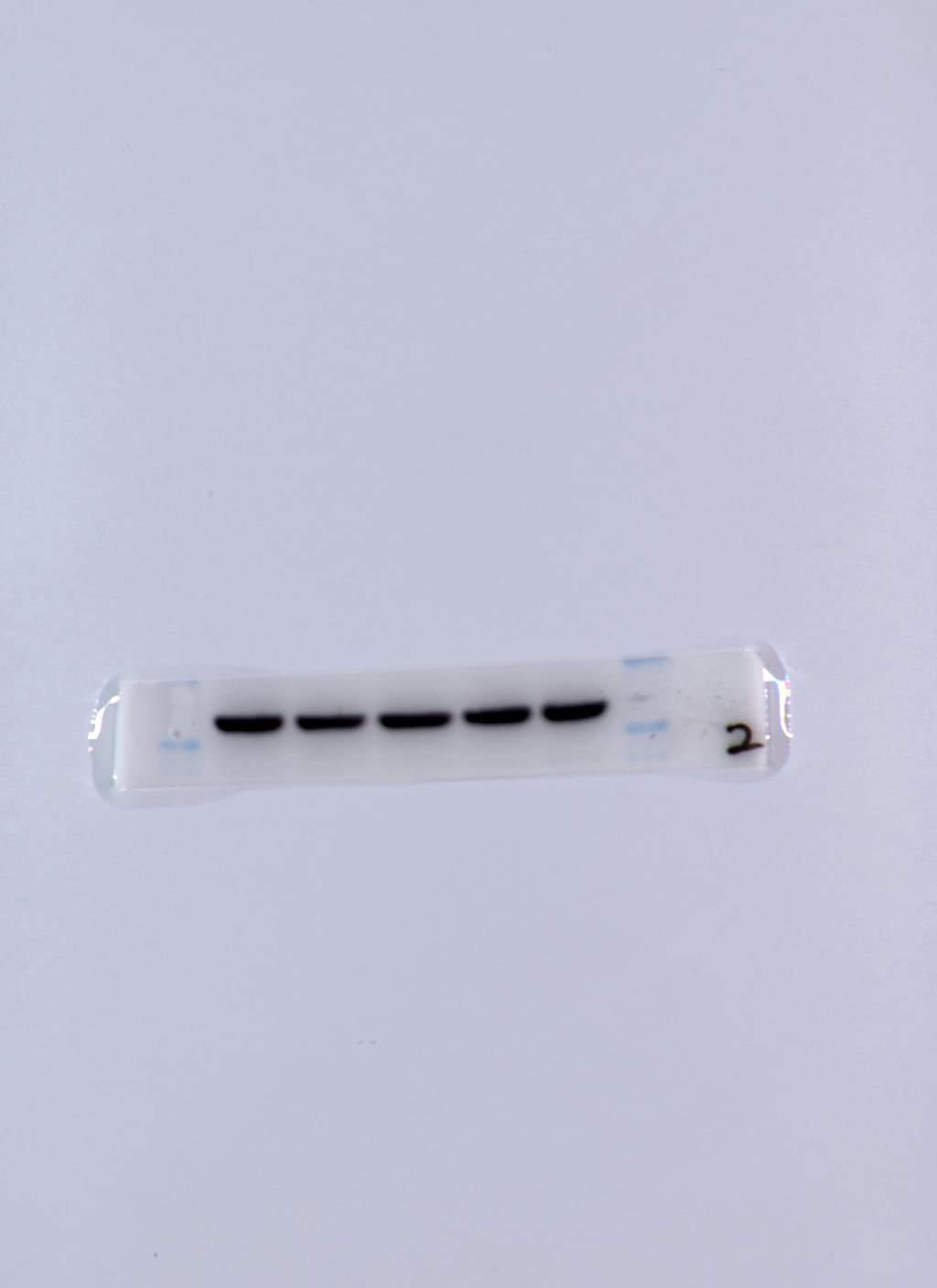

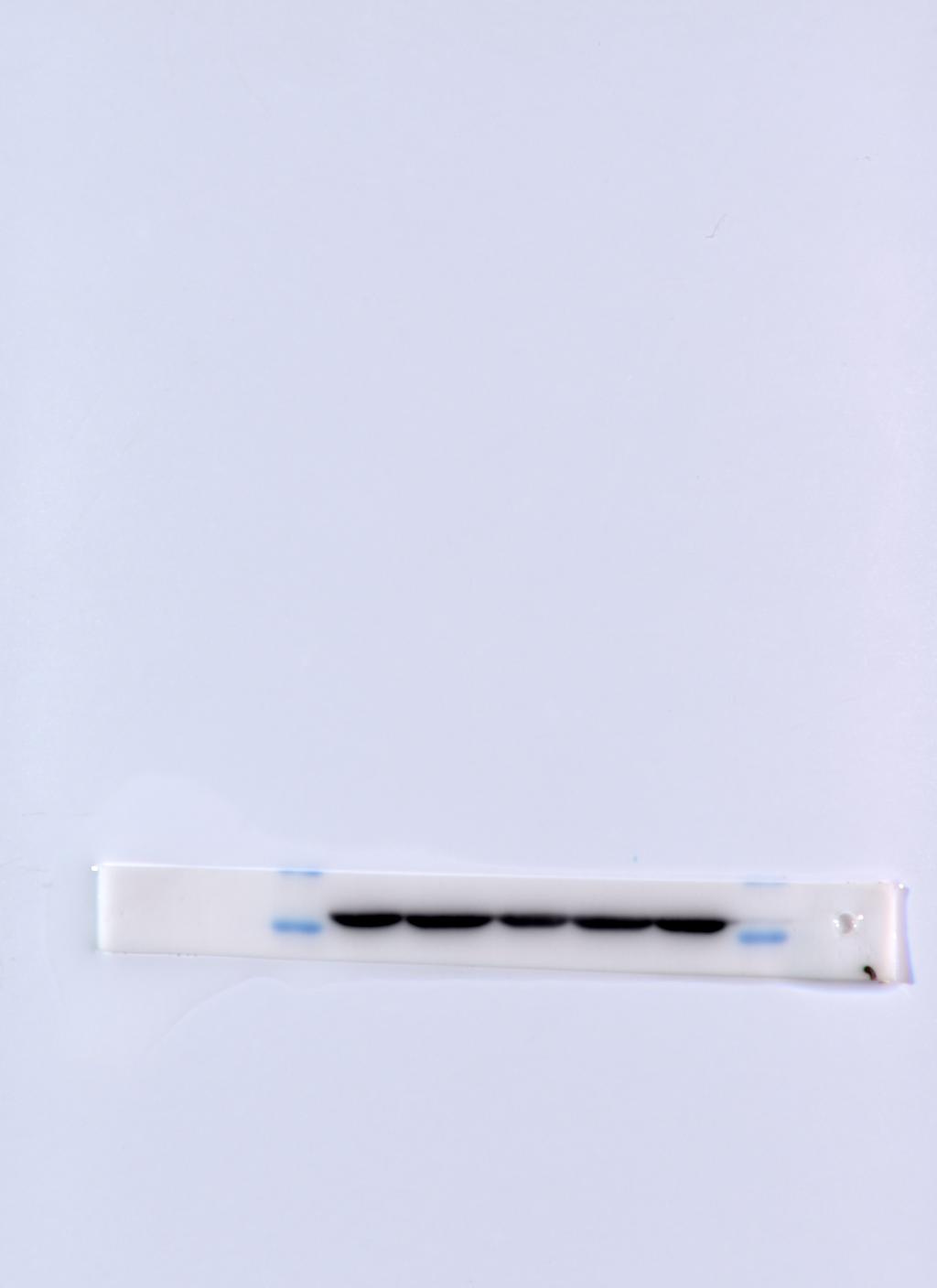


blot 2

blot 3

GLT-1

β-actin


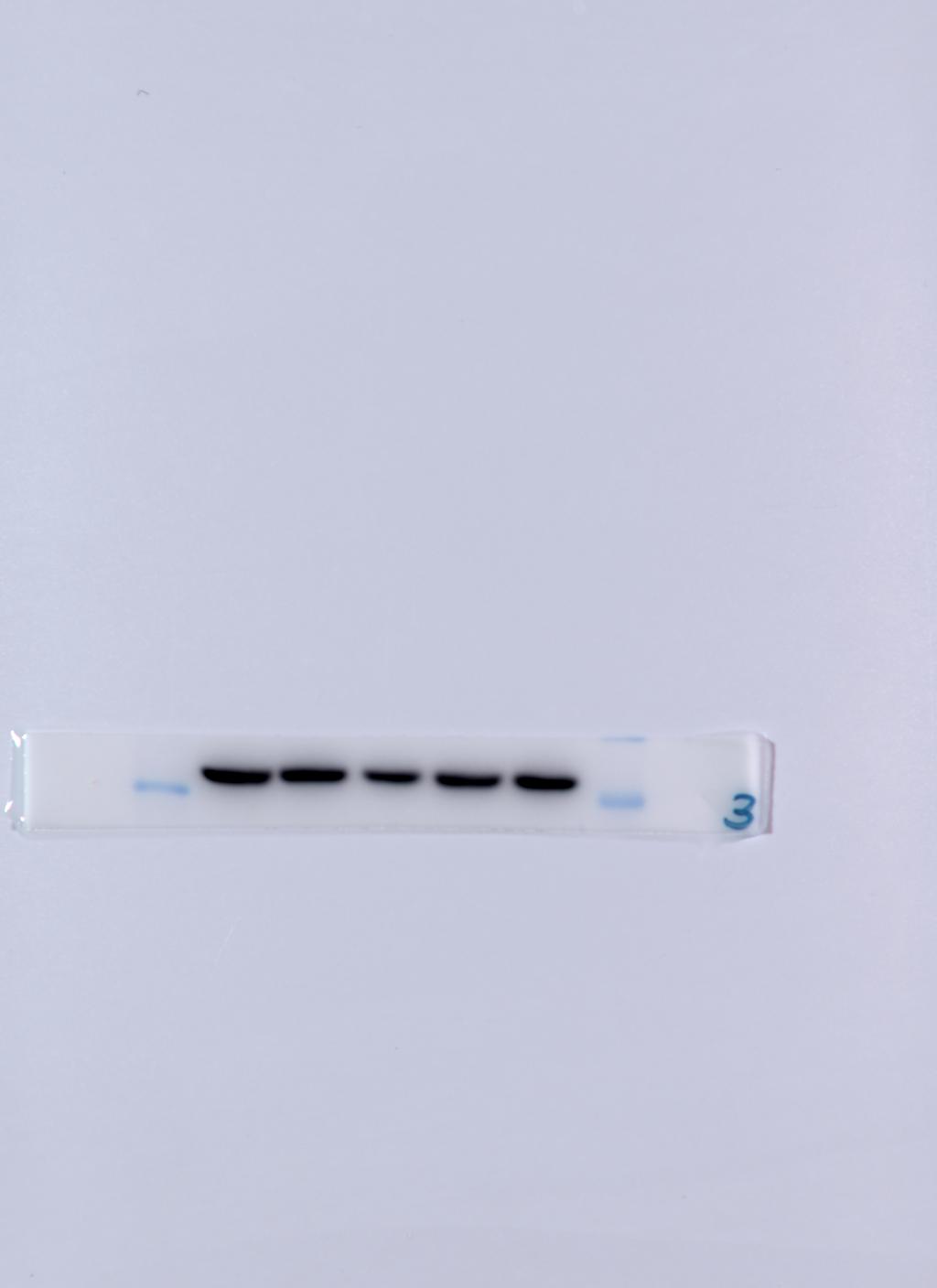

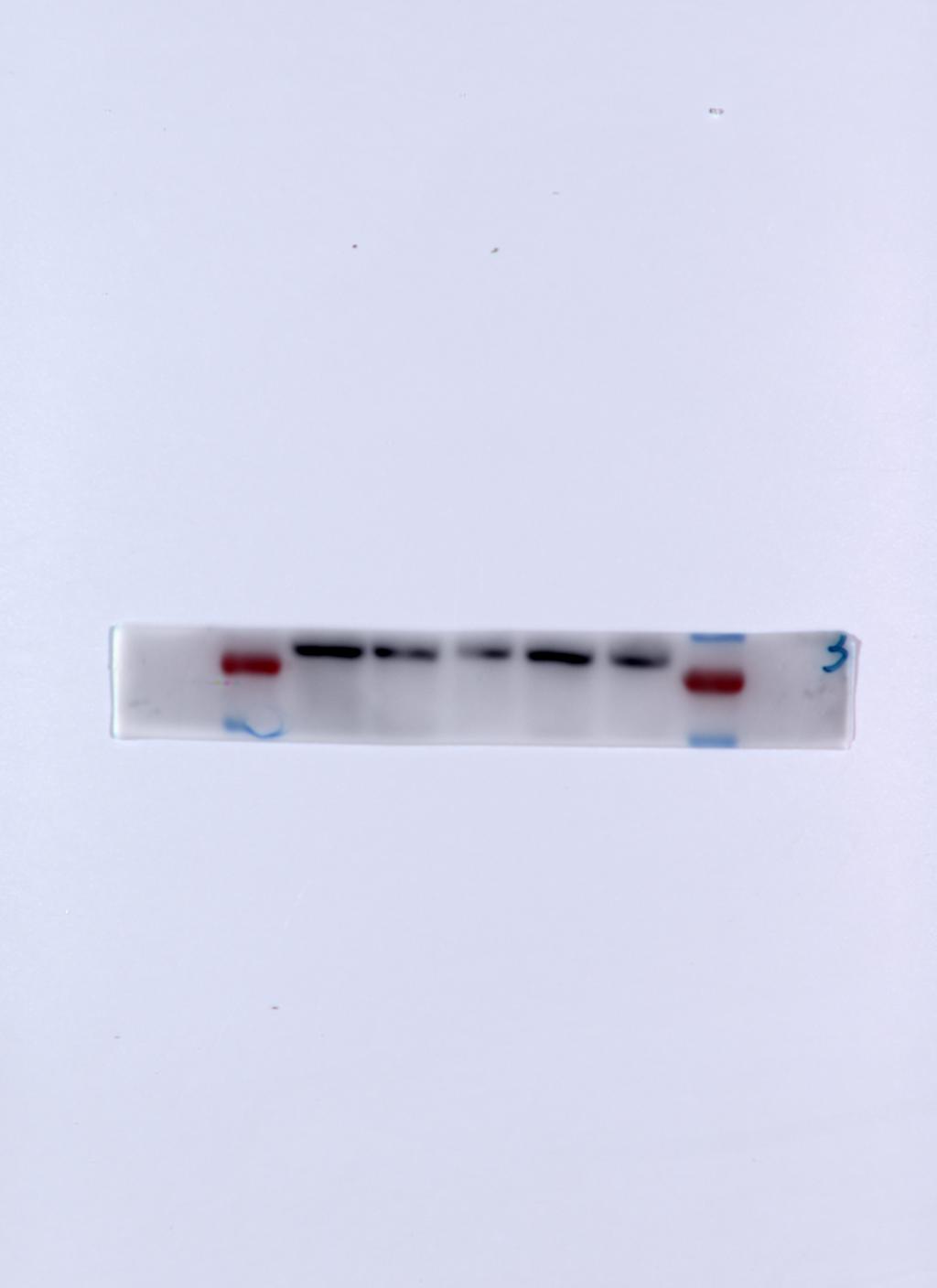


blot 1 (used in Fig 3H in the manuscript as representative blot)

**3. The western blot bands of xCT protein in each group**


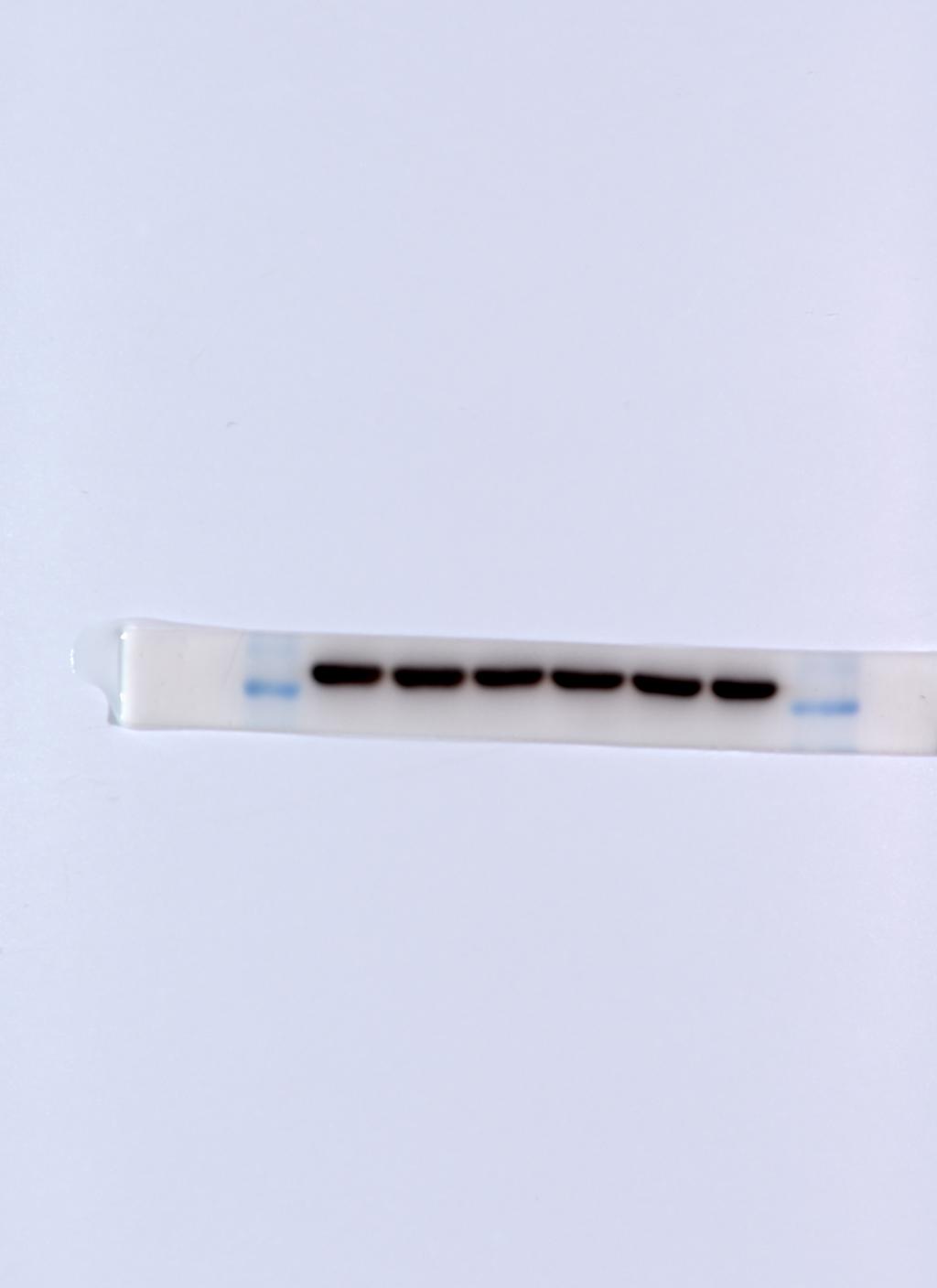


β-actin

The lane order from left to right on each blot is: C57, APP/PS1, GLT1^+/–^APP/PS1, Cef+APP/PS1, Cef+GLT1^+/–^APP/PS1.

blot 1 (used in Fig 5A in the manuscript as representative blot)

blot 2

xCT


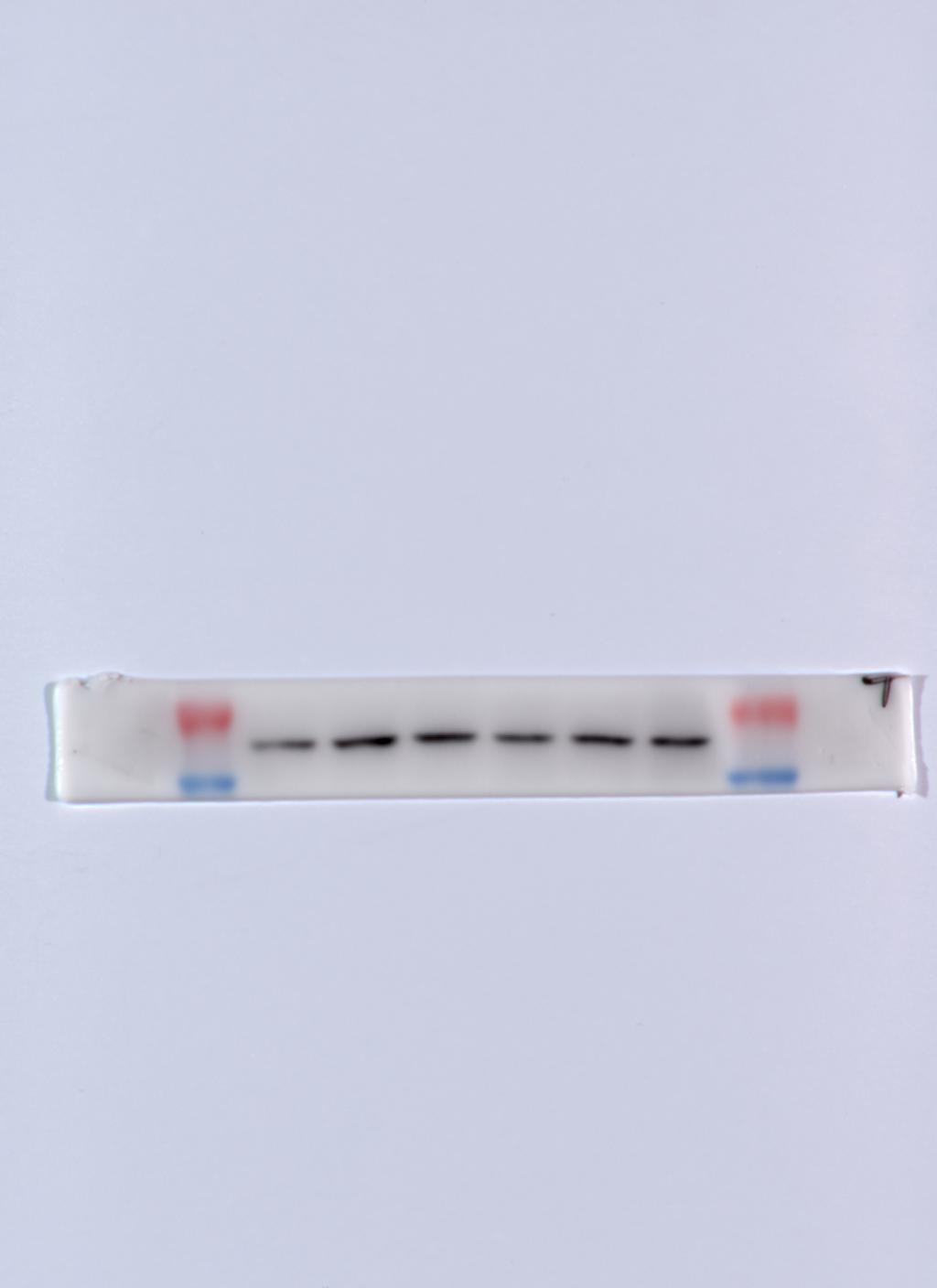

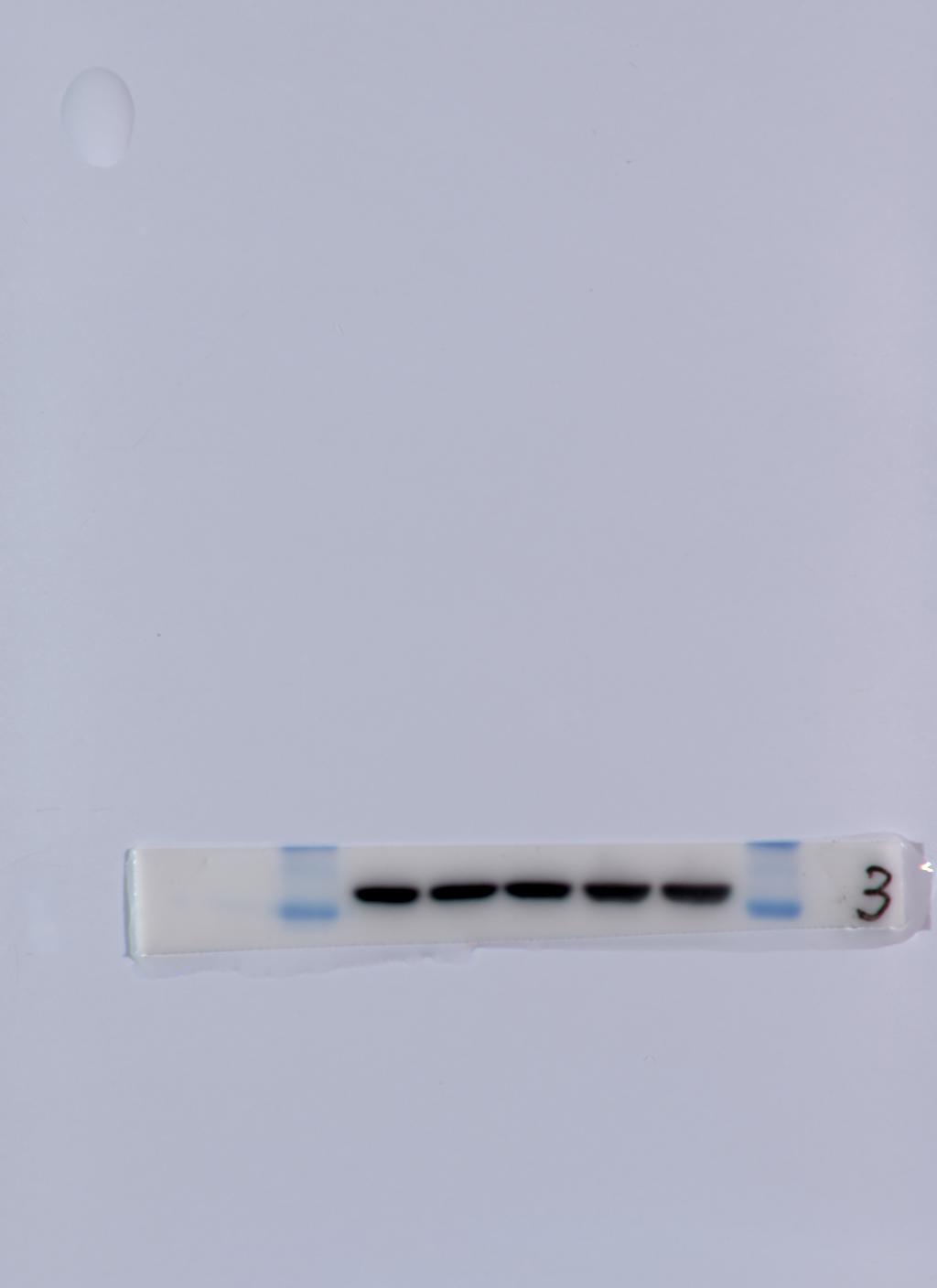

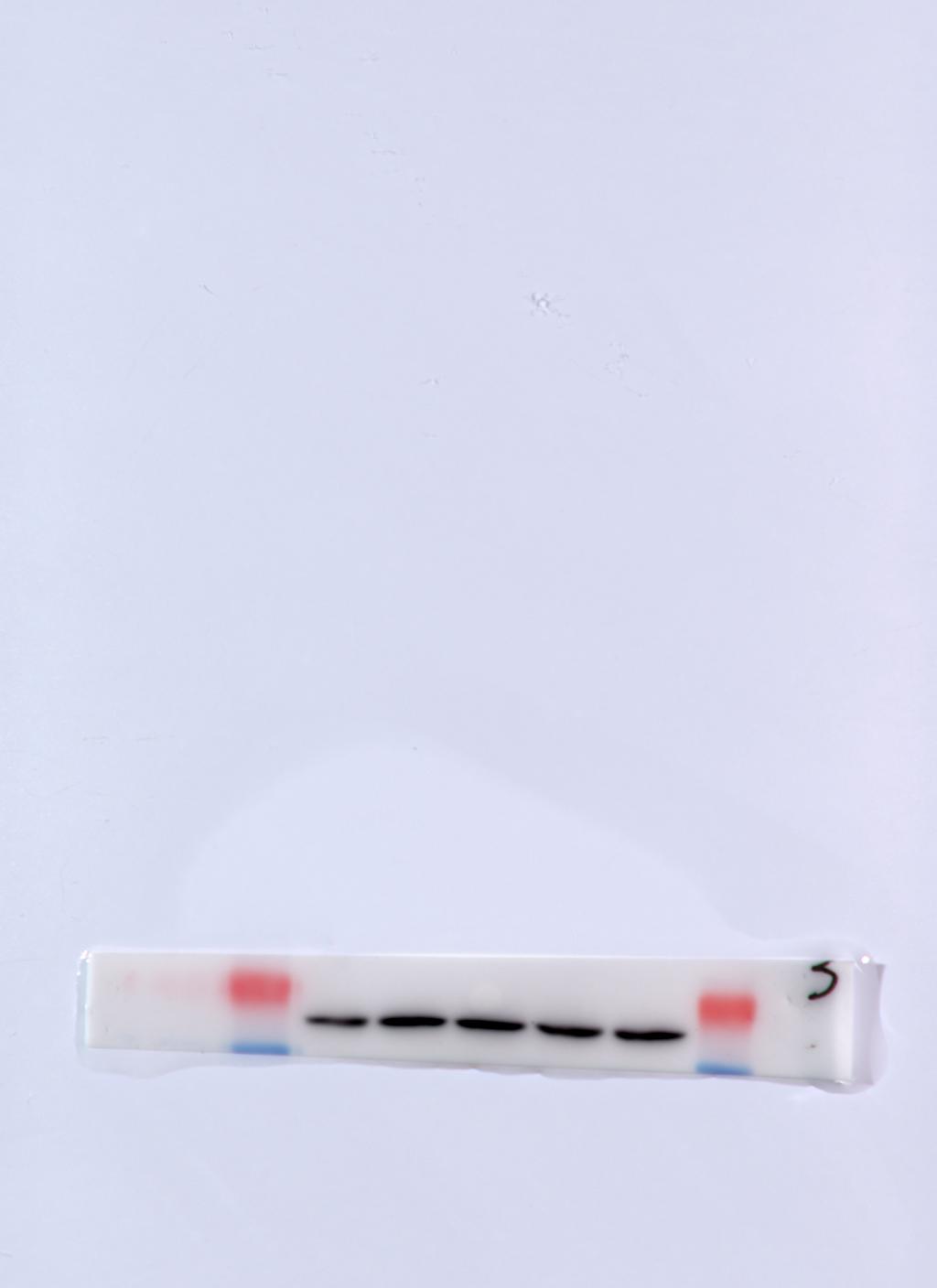

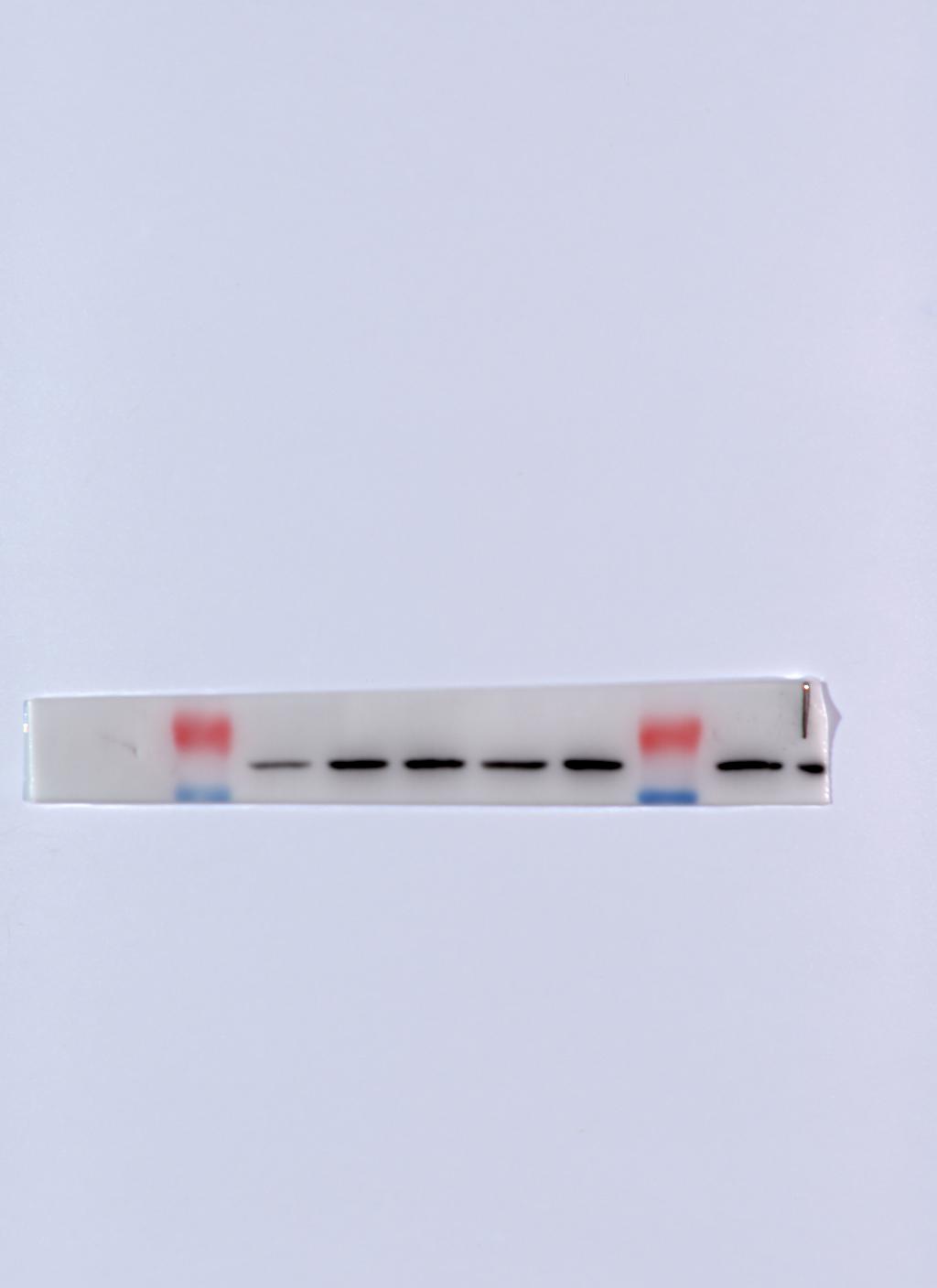

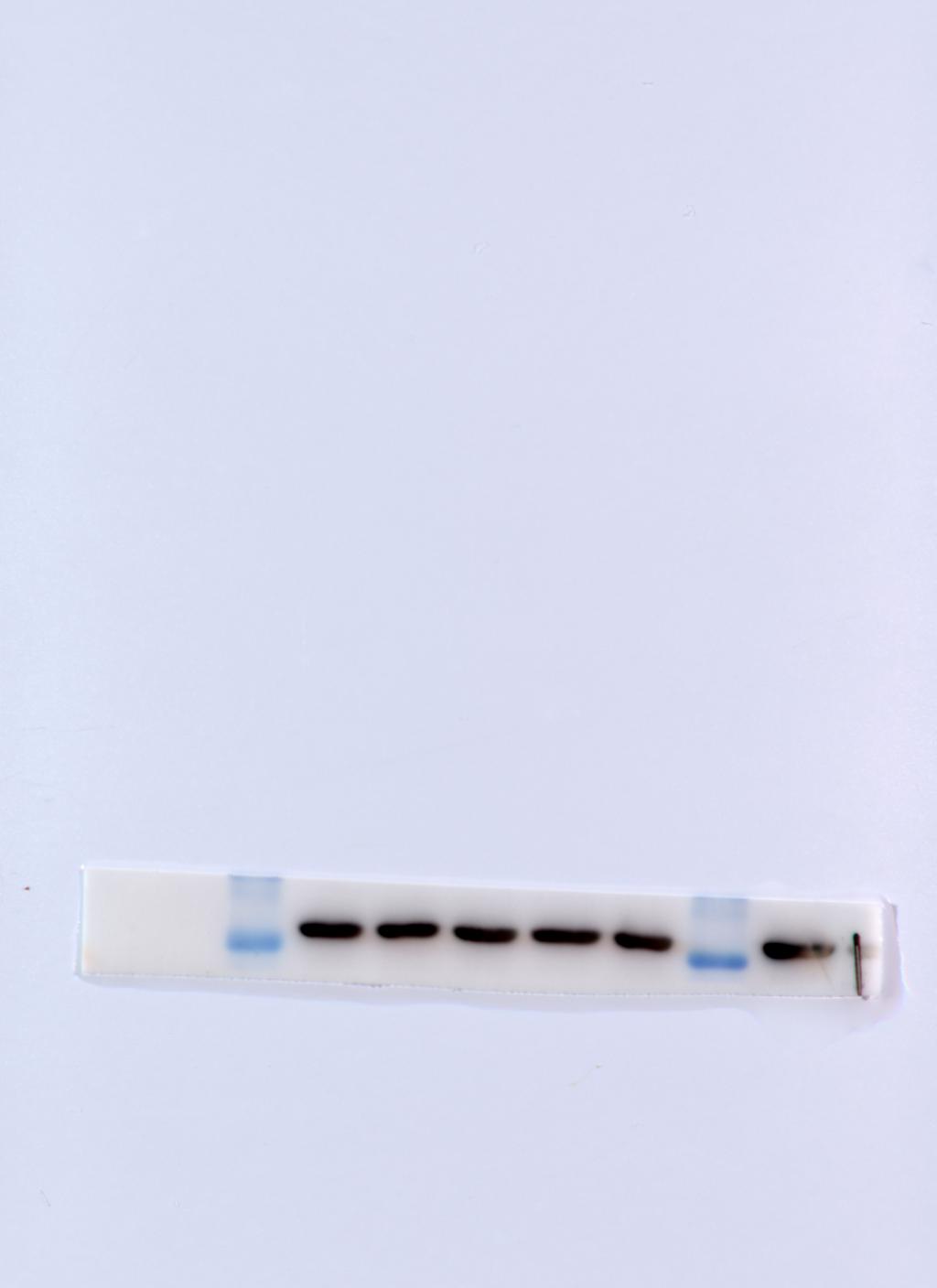

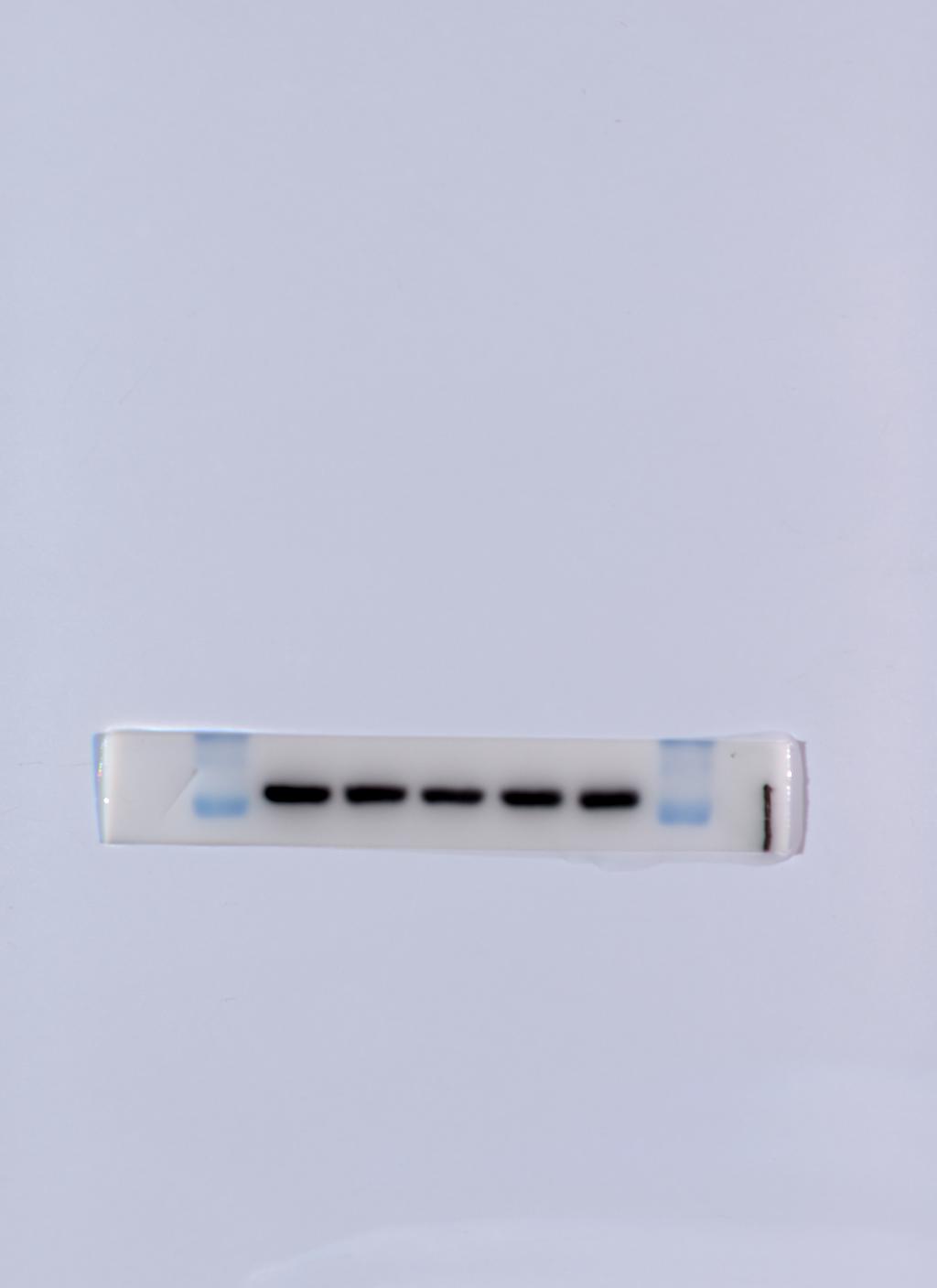

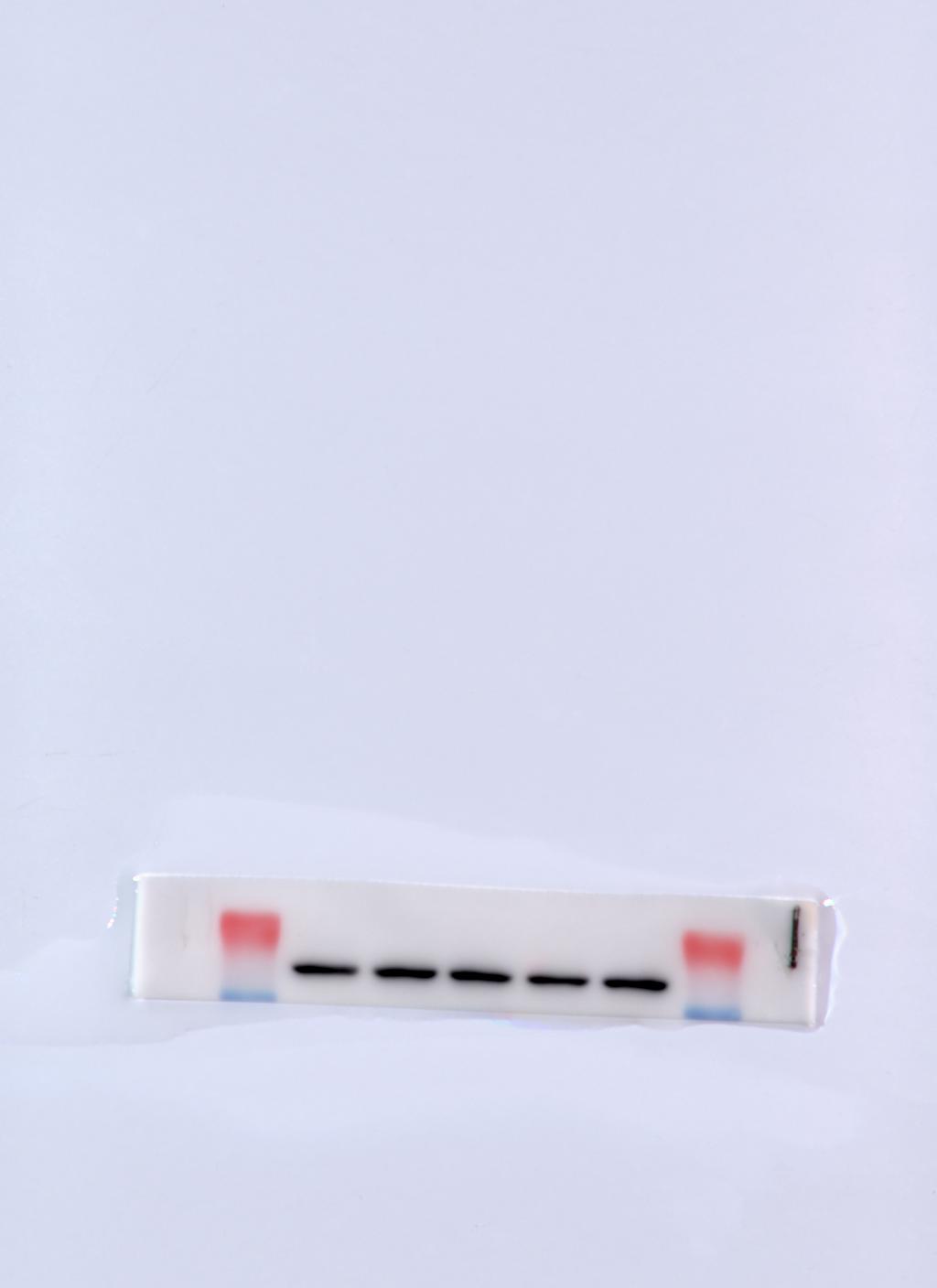

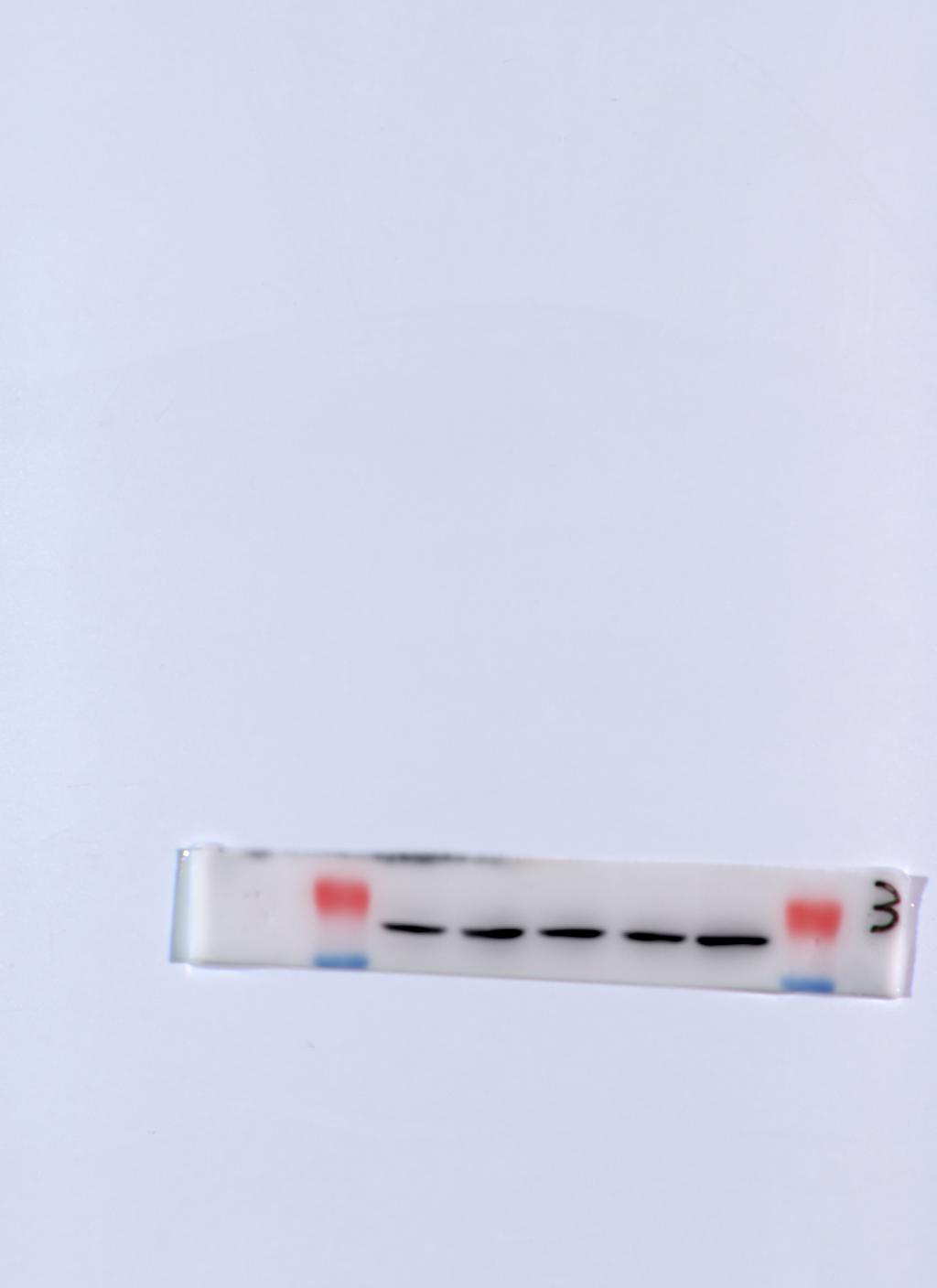

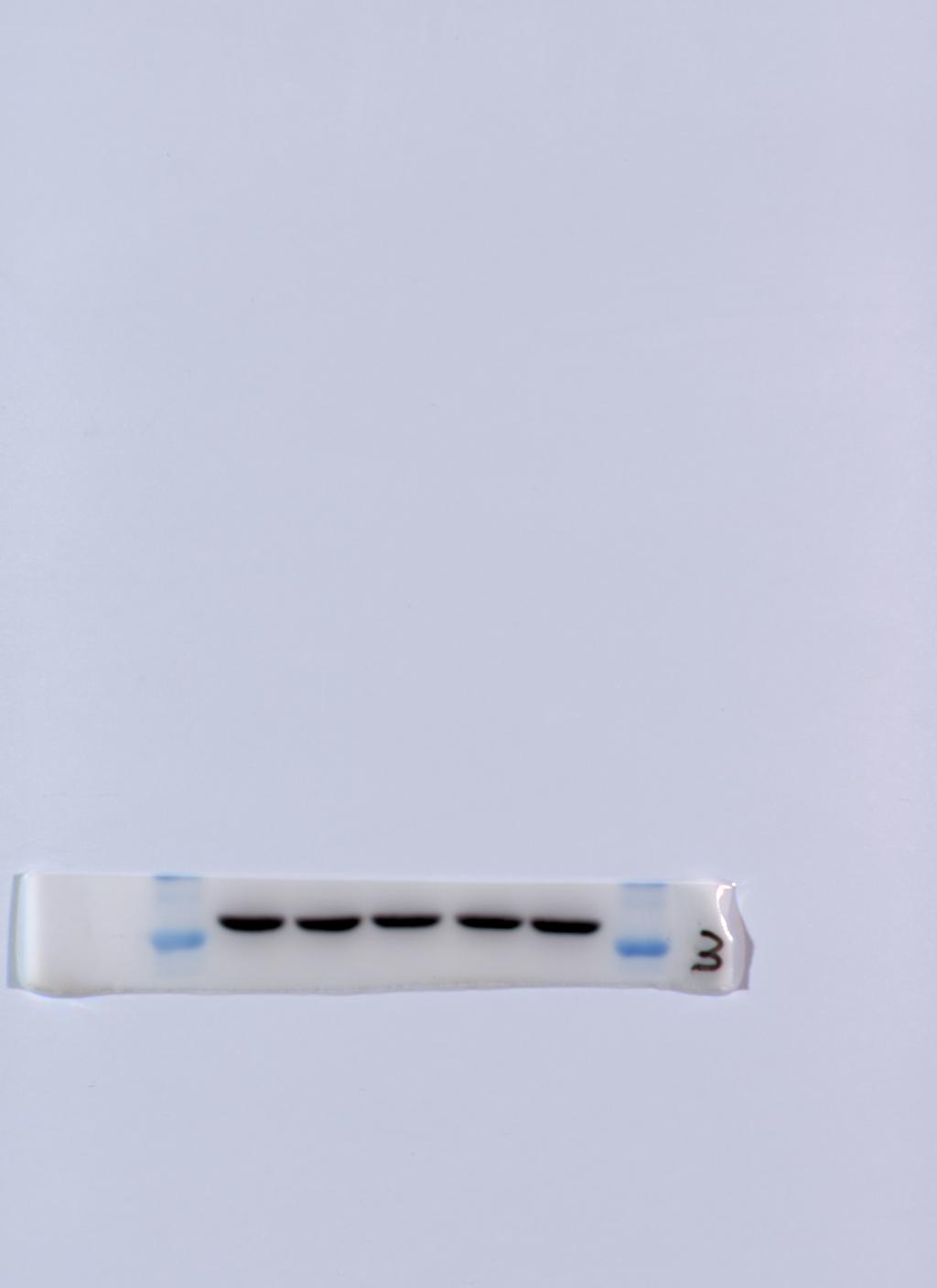


xCT

β-actin

xCT

β-actin

blot 4

blot 3

xCT

β-actin

xCT

β-actin


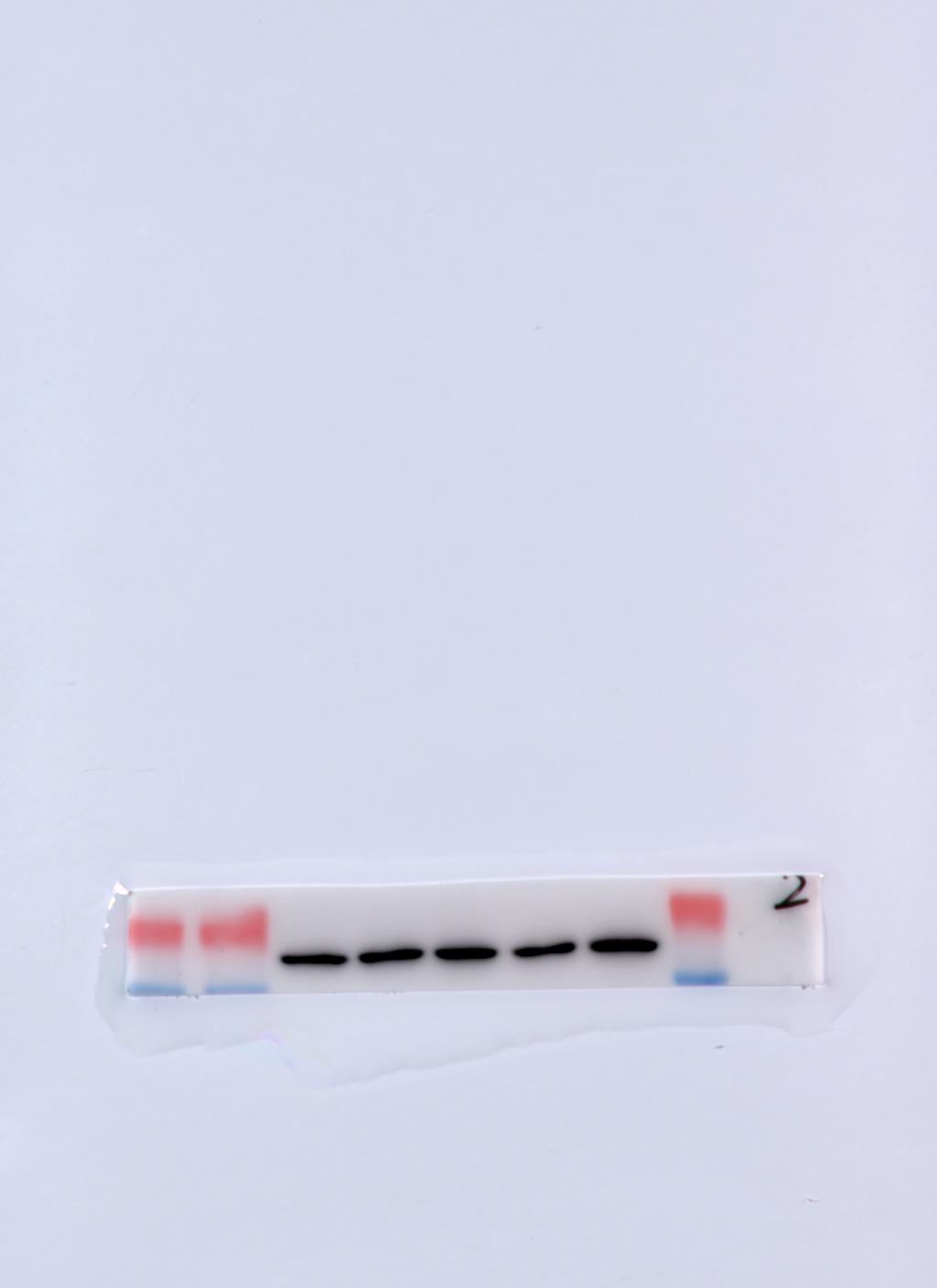

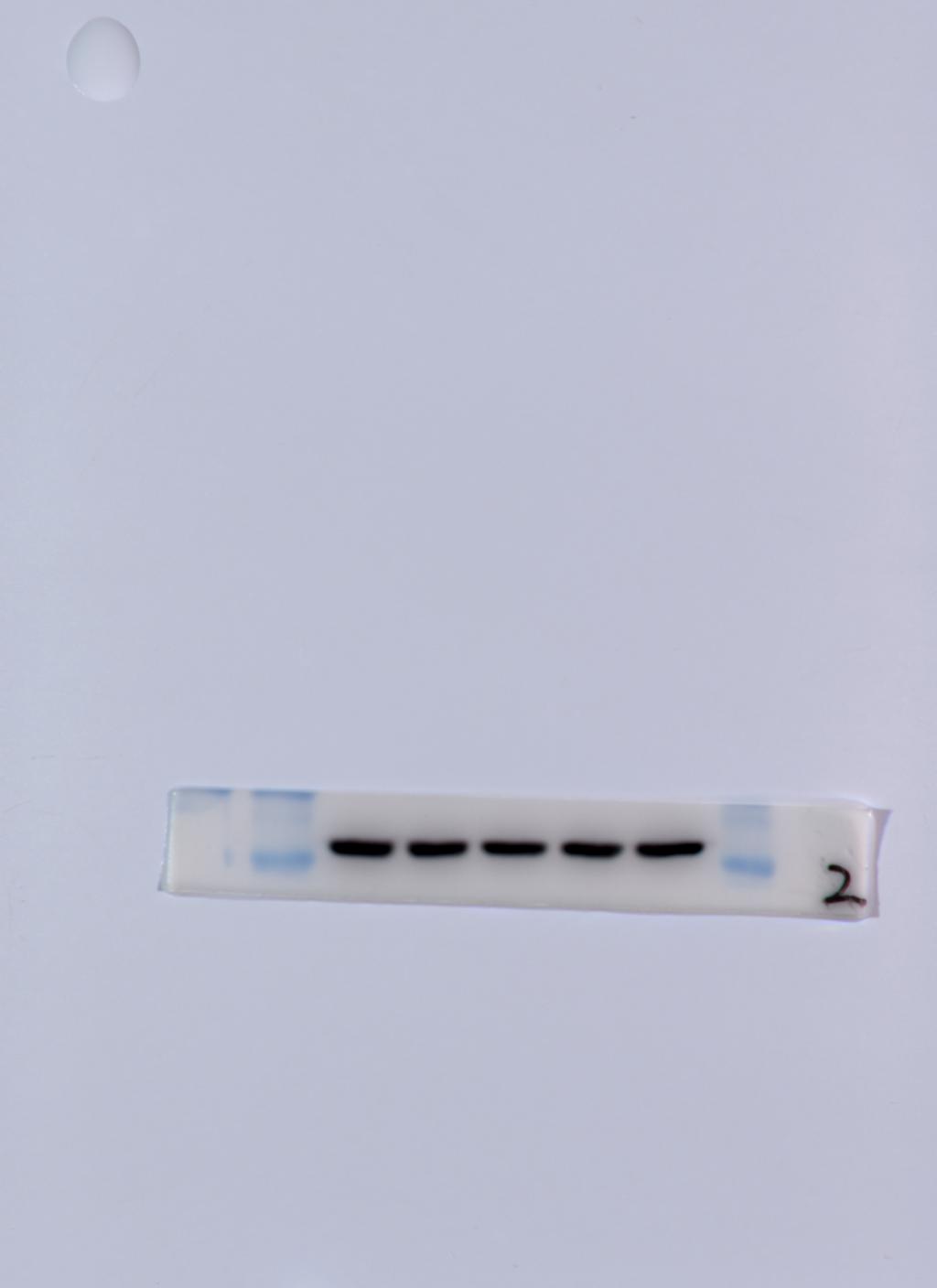


xCT

β-actin

blot 6

blot 5
