# Supplementary material for: GLT-1 Knockdown Inhibits Ceftriaxone-Mediated Improvements on Cognitive Deficits, and GLT-1 and xCT Expression and Activity in APP/PS1 AD Mice
Source: Front Aging Neurosci. 2020 Oct 6;12:580772. doi: 10.3389/fnagi.2020.580772 (PMC7574737; doi:10.3389/fnagi.2020.580772)
Supplement: Supplementary file 2 [file Table_2.DOC]

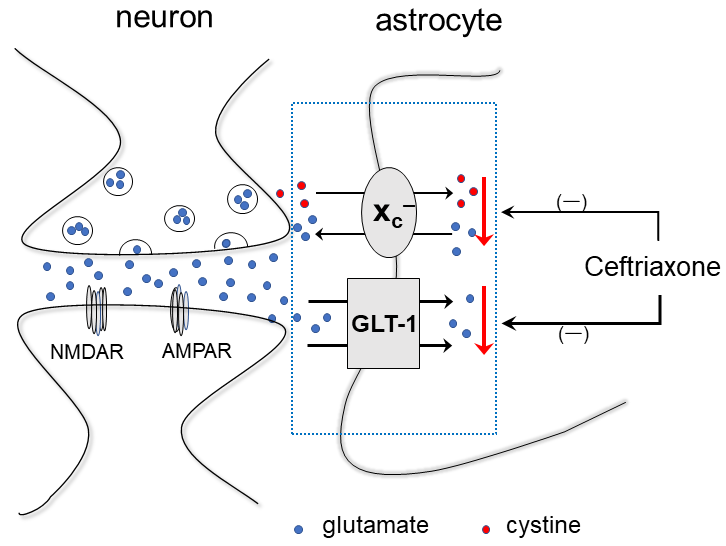


A diagram shows the findings of the present study. In APP/PS1 AD mice, the GLT-1 expression and uptake for glutamate was decreased, which induced an increase in extracellular glutamate concentration (indicated by dotted box). This circumstance might inhibit the activity of system xc^–^ and induces a decrease of intracellular cystine. Ceftriaxone restored the expression and uptake activity of GLT-1, which could promote the activity of system xc^–^ by decreasing glutamate concentration. These effects of ceftriaxone contribute to the ceftriaxone-mediated improvements in cognitive deficits in APP/PS1 AD mice.
